# Genome-Wide Investigation of the Phospholipase C Gene Family in *Zea mays*

**DOI:** 10.3389/fgene.2020.611414

**Published:** 2021-01-12

**Authors:** Jiantang Zhu, Yuanyuan Zhou, Jiale Li, Hui Li

**Affiliations:** School of Biological Science and Technology, University of Jinan, Jinan, China

**Keywords:** maize, PLC, genome-wide, expression pattern, stress, association analysis

## Abstract

Phospholipase C (PLC) is one of the main hydrolytic enzymes in the metabolism of phosphoinositide and plays an important role in a variety of signal transduction processes responding to plant growth, development, and stress. Although the characteristics of many plant PLCs have been studied, *PLC* genes of maize have not been comprehensively identified. According to the study, five phosphatidylinositol-specific PLC (PI-PLC) and six non-specific PLC (NPC) genes were identified in maize. The PI-PLC and NPC genes of maize are conserved compared with homologous genes in other plants, especially in evolutionary relationship, protein sequences, conserved motifs, and gene structures. Transient expression of ZmPLC-GFP fusion protein in *Arabidopsis* protoplast cells showed that *ZmPLCs* are multi-localization. Analyses of transcription levels showed that *ZmPLCs* were significantly different under various different tissues and abiotic stresses. Association analysis shown that some *ZmPLCs* significantly associated with agronomic traits in 508 maize inbred lines. These results contribute to study the function of ZmPLCs and to provide good candidate targets for the yield and quality of superior maize cultivars.

## Introduction

Phospholipids are important basic structural components of biological membranes and also as key signaling components responding to the plant development and various environmental stresses (Pokotylo et al., 2013). Phospholipids could be degraded into various products, such as diacylglycerol (DAG), phosphatidic acid (PA), free fatty acids (FFAs), and lysophospholipids (LPLs) by phospholipases, which include phospholipase C (PLC), phospholipase D (PLD), and phospholipase A (PLA) (Tuteja and Sopory, 2008; Hong et al., 2016). Among them, PLC is recognized as an important lipid hydrolase in animals and plants and has a profound effect on membrane lipid remodeling and intracellular signaling (Meldrum et al., 1991).

Based on different substrate affinities and cellular functions, plant PLCs have two different types: phosphatidylinositol-specific PLCs (PI-PLCs) and phosphatidylcholine-PLC (PC-PLC) (Kocourková et al., 2011; Pokotylo et al., 2014). PI-PLC hydrolyzes phosphoinositides to produce inositol 1,4,5-trisphosphate (IP_3_) and DAG, which may function as the second messengers (Berridge, 1987; Meldrum et al., 1991). IP_3_ could be quickly synthesized into hexakisphosphate (IP_6_) and trigger Ca^2+^ influx, while DAG could be phosphorylated by DAG kinase (DGK) and transformed into PAs (Wang et al., 2006). Unlike PI-PLC, PC-PLC, also known as non-specific PLC (NPC), preferentially hydrolyzes the common membrane phospholipids, for example, PC, phosphatidylethanolamine (PE), and phosphatidylserine (PS) (Kocourková et al., 2011).

In plants, two types of PLCs were composed of many gene members. For example, *Arabidopsis* contains nine PI-PLCs and six NPCs, whereas there are four PI-PLCs and five NPCs in rice, respectively (Nakamura et al., 2005; Zheng et al., 2012; Singh et al., 2013). In general, a typical plant PI-PLC enzyme structurally contains two domains at least, catalytic PI-PLC-X domain and PI-PLC-Y domain, which are necessary for PI-PLC to function as phosphoesterase (Hicks et al., 2008). Furthermore, the X and Y domains could form together a distorted triose phosphate isomerase (TIM) barrel structure, which contains the active-site residues (Chen et al., 2011). In addition, PI-PLCs have a C-terminal Ca^2+^/phospholipid binding C2 domain and an N-terminal EF hand domain involved in calcium binding (Chen et al., 2011). Some plant NPCs contain a putative signal peptide at the N-terminus, while all NPCs have a phosphoesterase domain, which is necessary for the function of esterase, such as NPCs and acid phosphatases (Wimalasekera et al., 2010). Generally, the phosphoesterase domain contains two highly conserved motifs, ENRSFDxxxG and TxPNR, and two other invariable motifs, DExxGxxDHV, and GxRVPxxxxxP (Pokotylo et al., 2013).

Members of the plant PLC family play important roles in various biological processes, for example, plant growth, development, and stress response. *AtPI-PLC2* is required for female gametogenesis and embryo development in *Arabidopsis*, and loss of *AtPI-PLC2* resulted in defective male and female gametophyte development (Li et al., 2015; Di Fino et al., 2017). Similarly, *AtNPC2* and *AtNPC6* are involved in gametophyte and embryo development and glycerolipid metabolism in the flower buds (Ngo et al., 2018), while *AtNPC3* and *AtNPC4* have an important regulatory role in root development (Wimalasekera et al., 2010). In addition, plant *PLCs* have also been confirmed to participate in a variety of tolerances to abiotic or biotic stresses. *Arabidopsis AtPI-PLC* members, except *AtPI-PLC2*, could be induced under various abiotic stresses such as salinity, drought, and cold (Tasma et al., 2008). Overexpression of *Brassica napus PI-PLC2* in canola induces significant changes in the expression of stress-related genes and enhances drought tolerance (Georges et al., 2009). Overexpression of *ZmPI-PLC1* enhanced the grain yield of maize under drought conditions, while suppression of *ZmPI-PLC1* had an opposite effect (Wang et al., 2008). Knockout of *AtNPC4* in *Arabidopsis* could increase sensitivity to salt stress in root elongation, seedling biomass, and seed germination, while *AtNPC5* expression could be significantly upregulated under salt stress and positively regulate the development of lateral root under salt stress (Kocourková et al., 2011).

In this research, the PLC-encoding genes including PI-PLC and NPC were identified in the maize genome. The *ZmPLCs* have been analyzed in detail, including phylogenetic relationships, gene structures, conserved motifs, and subcellular localization. The transcription levels of the *ZmPLCs* were determined by qRT-PCR in different tissues and various abiotic stresses. In addition, the *ZmPLC* genes with quantitative trait loci (QTLs) associated with agronomic traits. Furthermore, the results presented herein provide valuable clues for studying the functions of *ZmPLCs* in response to the growth, development, and stress responses of maize.

## Materials and Methods

### Genome-Wide Identification of the *PLC* Genes in Maize

To identify putative PLC proteins, the hidden Markov models (HMMs) of the two characteristic domains of a PLC protein from PFam (http://pfam.sanger.ac.uk/), PI-PLC-X (PF00388), and PI-PLC-X (PF00387) were used as query sequences in local HMM-based searches, setting *E-*values < 0.01. In addition, to identify ZmPLC proteins that might have been missed through HMM searching, ZmPLC sequences were further identified using the previously reported *Arabidopsis* PLC protein sequences from the Maize Genome Database (https://www.maizegdb.org) and Phytozomev12.0 (http://www.phytozome.net) under the *E-*value cutoff 0.1. The matched sequences were subjected to SMART (http://smart.embl.de/) analyses to detect the presence and number of the PLC domain. Therefore, 11 independent *ZmPLC* genes were identified in maize. The chromosomal location image was mapped by MapInspect software. The ExPASy (https:// web.expasy.org/protparam/) was performed to calculate the molecular weight (MW) and the theoretical isoelectric point (PI).

### Phylogenetic Analysis and Synteny Analysis

The multi-species PLC sequences, including 11 ZmPLCs from *Zea mays*, 15 AtPLCs from *Arabidopsis thaliana*, nine OsPLCs from *Oryza sativa*, 22 GhPLCs from *Gossypium hirsutum*, eight BdPLCs from *Brachypodium distachyon*, 10 SbPLCs from *Sorghum bicolor*, and 19 GmPLCs from *Glycine max* (Supplementary File 1), were constructed into a phylogenetic tree using the neighbor-joining (NJ) method in MEGA7.1 (Tamura et al., 2011). *ZmPLCs* were named basing on the phylogenetic relationship with *AtPLCs* and *OsPLCs*. Syntenic gene pairs among *Zea mays* and between *Arabidopsis thaliana* and *Oryza sativa* were identified using the TBtools (Chen et al., 2020).

### Analyses of Gene Structures, *cis*-Acting Elements, and Motifs

The exon/intron structures of *PLCs* were analyzed by Gene Structure Display Server (GSDS) (http://gsds.cbi.pku.edu.cn/). The genomic sequences 2,000 bp upstream of *ZmPLCs* predicted the *cis*-acting elements using PlantCARE software (http://bioinformatics.psb.ugent.be/webtools/plantcare/html/?tdsourcetag=s_pcqq_aiomsg). The conserved motifs were analyzed with MEME (http://meme.sdsc.edu/meme4_3_0/intro.html).

### Subcellular Localization of ZmPLCs

In order to confirm the subcellular localization, the coding sequences of selected *ZmPLC* genes were amplified using gene-specific primers. Then, ZmPLCs minus the stop codons were cloned and inserted into pBI221:eGFP, and the corresponding expression vectors were introduced into *Arabidopsis* protoplasts. The green fluorescent protein (GFP) fluorescence was excited with a confocal laser scanning microscope LSM 800 (Zeiss).

### Plant Growth Conditions and Treatments

The maize inbred line W22 (from Huazhong Agricultural University) was used for all the experimental treatments. The seeds were sterilized with 70% ethanol for 5 min and then washed three times with sterile water. The seedlings were grown in a Hoagland solution in a greenhouse with a regime of 16 h light/8 h dark and at 28°C. When the maize seedlings were raised to the three-leaf stage, seedlings were selected for stress treatments according to Lin et al. (2014), including 20% PEG 6000 for drought stress, 200 mM NaCl for salt stress, 4°C for cold stress, and 20 μmol/L Cu^2+^ for heavy metal stress. The leaves were collected at different points in time after treatment. Each treatment consisted of three replicates. Adult plants were grown in the field, and then all organs and tissues were harvested. The root was harvested at the three-leaf stage; the stem and leaf were collected at five fully extended leaves; the silk, cob, and anther were harvested at 13 extended leaves; the kernel was harvested at 10 days after pollination (DAP). All materials were immediately frozen in liquid nitrogen after harvesting and stored at −80°C prior to RNA isolation.

### Expression Analysis and Quantitative Real-Time PCR

For quantitative real-time PCR, total RNAs were extracted from various maize tissues with the RNAprep Pure Plant Kit (Tiangen, Beijing, China). According to supplier instructions, total cDNA was synthesized using PrimeScript^TM^ RT Reagent Kit with gDNA Eraser (Tiangen, Beijing, China). The Primer Premier 5.0 was used to design the primers for qRT-PCR (Supplementary Table 1), and the maize TUB-ribosylation factor was selected as an internal control. The reaction was performed on Bio-Rad CFX Connect^TM^ using SYBR-Green to detect gene expression levels. For all qRT-PCR analyses, triplicate biological samples were collected. Data were analyzed using Bio-Rad CFX Manager software.

### Candidate Gene-Based Association Mapping of *ZmPLC* Family Members in Maize

Regional association tests between the single-nucleotide polymorphisms (SNPs) of candidate genes and the 17 traits agronomic, including plant height, ear height, tassel branch number, ear diameter, 100 grain weight, silking time, heading date, leaf number above ear, tassel main axis length, ear length, kernel width, cob weight, pollen shed, kernel weight, kernel number per row, ear leaf width, ear leaf length, were conducted in the 508 inbred lines (Fu et al., 2013). The genotype and phenotypes of the association panel were detected by two genotyping platforms, resulting in 550,000 high-quality SNPs (Yang et al., 2011; Li et al., 2013), and only SNPs within the range of 100 kb upstream and downstream of candidate genes were used. The association analysis was estimated using a mixed linear model (MLM) incorporated in TASSEL V5.0 (Bradbury et al., 2007). *P* ≤ 0.05 was considered the significance threshold.

## Results

### Identification and Characterization of *PLCs* in Maize

Eleven ZmPLCs, including five PI-PLC and six NPC sequences, were identified in the maize genome according to their domain structures (Supplementary Figure 1A). The ZmPI-PLC group contains five members: ZmPI-PLC1, ZmPI-PLC2, ZmPI-PLC3a, ZmPI-PLC3b, and ZmPI-PLC4. The ZmNPC group has six members: ZmNPC1a, ZmNPC1b, ZmNPC2, ZmNPC3, ZmNPC4, and ZmNPC5. Domain analysis showed that the five ZmPI-PLCs contained the catalytic PI-PLC-X, PI-PLC-Y domain, and Ca^2+^/phospholipid binding C2 domain, whereas an EF hand-like motif was found only in ZmPI-PLC4 (Supplementary Figure 1A). Six ZmNPCs had a phosphoesterase domain, which contains two highly conserved motifs, ENRSFDxxxG and TxPNR, and two invariable motifs, DExxGxxDHV and GxRVPxxxxxP (Supplementary Figure 1B). The gene identification (ID), gene name, open reading frame size, exon number, length, molecular weight, and isoelectric point of *ZmPLCs* were shown in Table 1. Especially, the number of amino acids (aa) in ZmPLCs of maize is comparable, with ZmPI-PLCs ranging from 586 to 606 aa and ZmNPCs ranging from 489 to 542 aa (ZmNPC1b is the only exception with 259 aa) (Table 1).

**Table 1:** General information of *PLC* genes in maize.

**Gene Name**	**Gene ID (AGPv4)**	**Gene ID (AGPv3)**	**Chromosome Location**	**ORF (bp)**	**Exon No**.	**Protein (aa)**	**PI**	**MW (KD)**	**Type**	**Sub-Localization Prediction**
*ZmPI-PLC1*	Zm00001d007229	GRMZM2G129238	chr2:225211077-225213907 (+)	1,764	7	587	6.46	66.97	PI-PLC	Chloroplast
*ZmPI-PLC2*	Zm00001d028746	GRMZM2G114354	chr1:45171565-45179033(+)	1,770	9	589	5.98	66.52	PI-PLC	Cytoplasm
*ZmPI-PLC3a*	Zm00001d014903	GRMZM5G889467	chr5:67438945-67442847(+)	1,761	9	586	6.40	65.68	PI-PLC	Chloroplast
*ZmPI-PLC3b*	Zm00001d014906	GRMZM2G137435	chr5:67681040-67684923(+)	1,761	9	586	6.40	65.85	PI-PLC	Chloroplast
*ZmPI-PLC4*	Zm00001d047447	GRMZM2G157760	chr9:130364956-130370673(+)	1,821	6	606	5.94	68.23	PI-PLC	Mitochondrion
*ZmNPC1a*	Zm00001d034714	GRMZM2G139041	chr1:300523648-300526448(+)	1,629	3	542	7.37	60.60	NPC	Chloroplast
*ZmNPC1b*	Zm00001d012924	GRMZM2G116876	chr5:2004892-2007704(+)	780	3	259	6.07	28.96	NPC	Nucleus
*ZmNPC2*	Zm00001d042057	GRMZM2G479112	chr3:148970593-148972670(+)	1,548	2	515	6.25	57.49	NPC	Mitochondrion
*ZmNPC3*	Zm00001d034875	GRMZM2G422670	chr1:304506320-304508029(+)	1,626	2	541	5.82	59.02	NPC	Cytoplasm
*ZmNPC4*	Zm00001d032907	–	chr1:242704706-242711363(+)	1,470	2	489	5.74	53.14	NPC	Cytoplasm
*ZmNPC5*	Zm00001d040205	GRMZM2G081719	chr3:32011584-32013797(+)	1,593	2	530	9.09	58.41	NPC	Chloroplast

*ID, identification; ORF, open reading frame; PI, isoelectric point; MW, molecular weight; aa, amino acid*.

### Phylogenetic Relationship, Gene Structure, and Conserved Motifs of *ZmPLCs* in Maize

**Figure 1:**
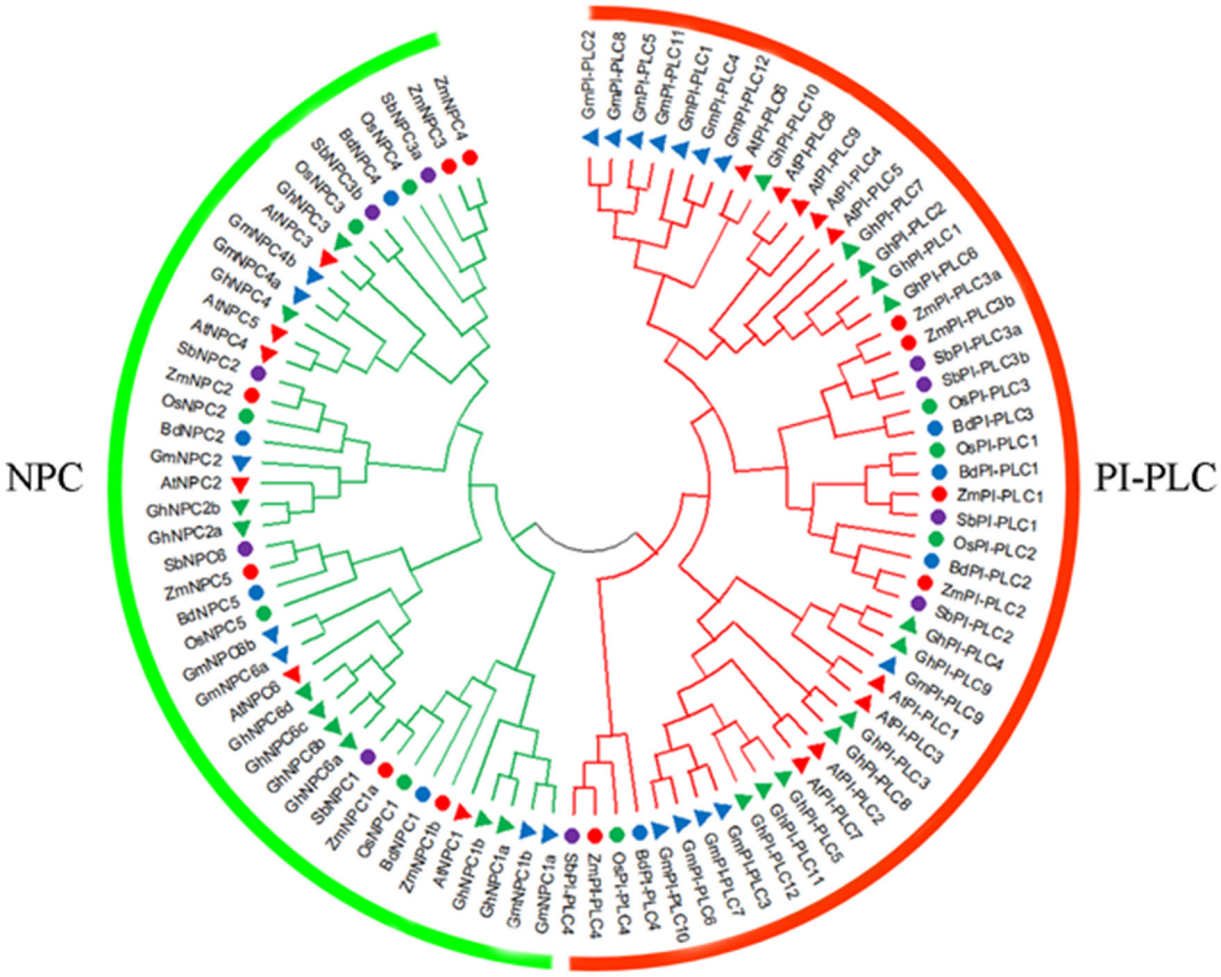
Phylogenetic analysis of the *PLC* family. The phylogenetic tree was made by MEGA 7.1 software using the neighbor-joining (NJ) method with bootstrapping analysis. The analysis involved 94 amino acid sequences from various plants, including *Arabidopsis thaliana* (At), *Glycine max* (Gm), *Oryza sativa* (Os), *Sorghum bicolor* (Sb), *Gossypium hirsutum* (Gh), *Brachypodium distachyon* (Bd).

To investigate the phylogenetic relationship of *PLC* among different species, a phylogenetic tree consisting of 11 ZmPLCs, 15 AtPLCs, nine OsPLCs, 19 GmPLCs, eight BdPLCs, 10 SbPLCs, and 22 GhPLCs was constructed using the NJ method (Figure 1). A total of 94 PLC protein sequences were classified into two subfamilies, named PI-PLC and NPC, based on the differences in the domain and phylogenetic relationships (Figure 1). The phylogenetic analysis showed that ZmPLCs shared high homology with those from other plants (Figure 1), especially ZmPI-PLC1, ZmPI-PLC2, ZmPI-PLC3a, and ZmPI-PLC3b made a separate small clade, while ZmPLC4 fell apart (Figure 1). While ZmNPC1a and ZmNPC1b; ZmNPC2; ZmNPC5; ZmNPC3 and ZmNPC4 made a separate small clade (Figure 1).

**Figure 2:**
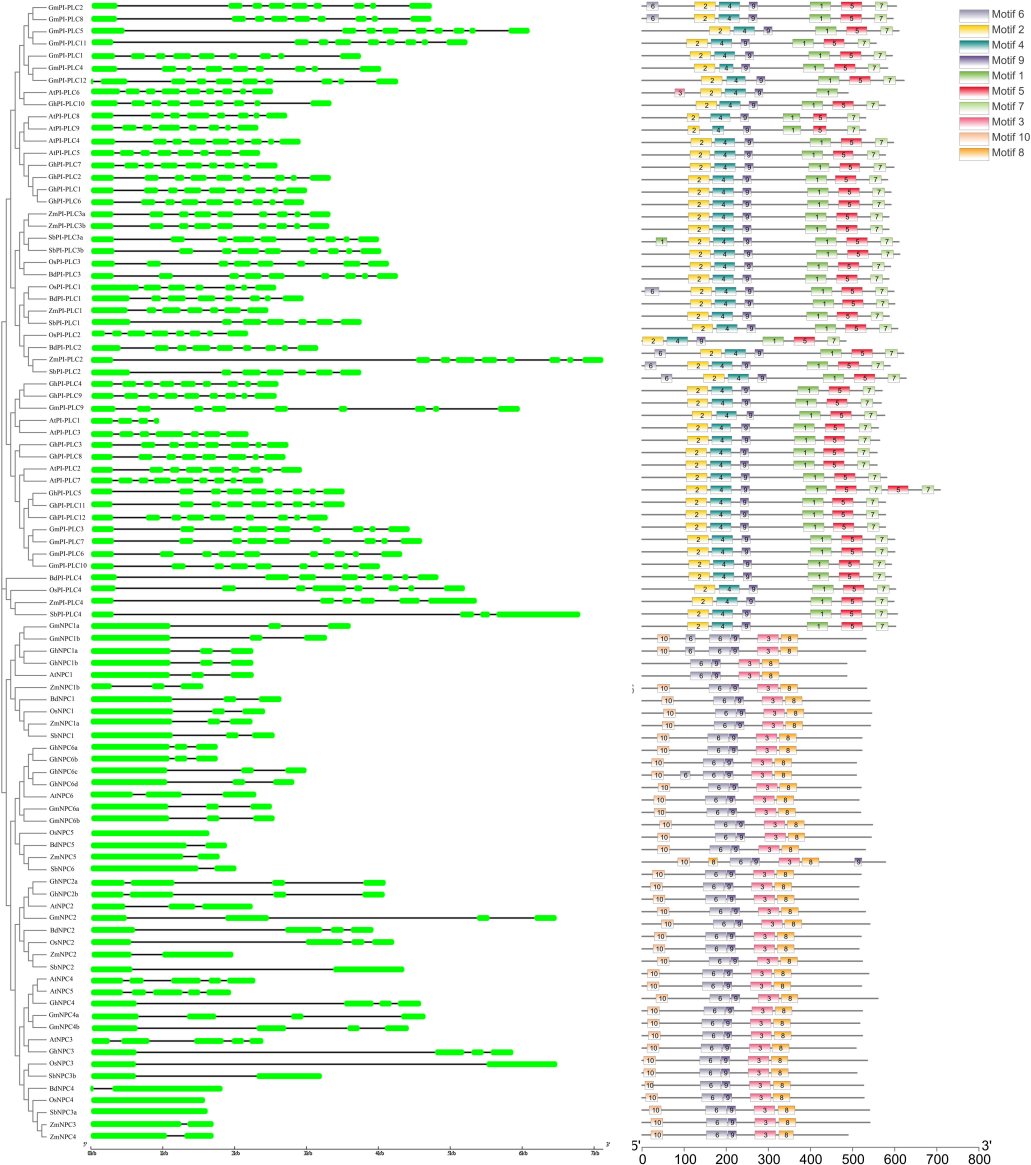
Phylogenetic relationships, gene structures, and conserved protein motifs of maize *PLC* family. Green boxes indicate exons, and black lines indicate introns. The motifs, numbered 1–10, are displayed in different colored boxes. The length of the protein can be estimated using the scale at the bottom.

Corresponding to the evolutionary relationship, analysis of the exon/intron structures of the *PLC* genes revealed that these genes are also divided into two different types: PI-PLC with an exon-rich clade (≥5 exons per gene) generally containing 5–10 exons, while NPC with an exon-poor clade (≤5 exons per gene) containing 1–5 exons (Figure 2). It is worth noting that a similar exon/intron pattern exists in each clade, for instance, most NPC1 genes contain three exons, and the vast majority of PI-PLC1 genes have seven exons (Figure 2).

To further study the characteristic regions of the PLC proteins, a total of 10 conserved motifs were identified in PLCs using the online MEME tool (Figure 2). Interestingly, the members of the same clade usually had similar structures and lengths in terms of domain. The group PI-PLC members had the conserved motifs 1, 2, 4, 5, 7, and 9, which are PI-PLC-X, PI-PLC-Y, and Ca^2+^/phospholipid binding C2 domain. While the conserved motifs 3, 6, 8, 9, and 10, which were annotated as the phosphoesterase domain, were found in group NPC family (Figure 2).

### Chromosomal Location of the *ZmPLCs* and Synteny Analysis of *PLCs* Among Several Different Species

**Figure 3:**
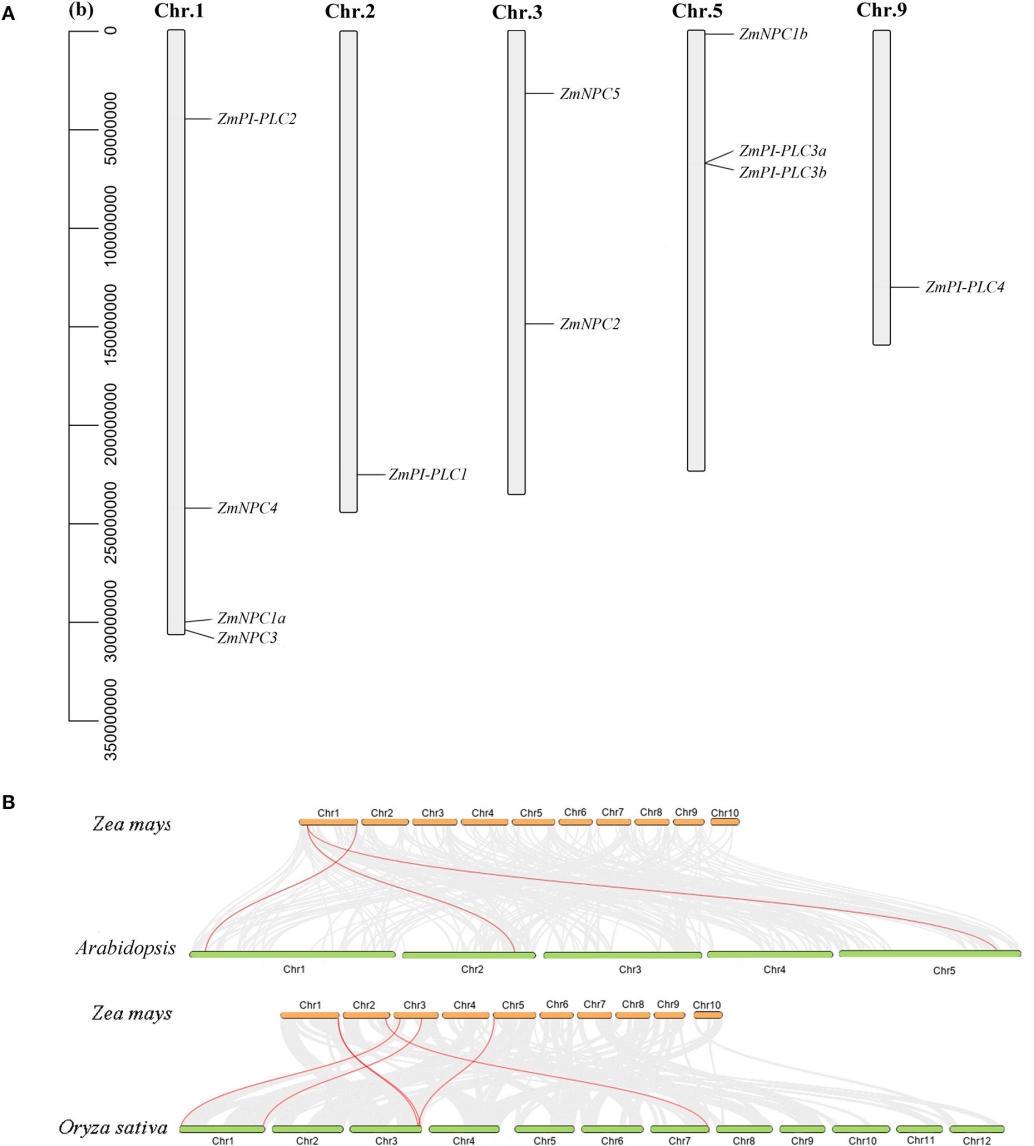
The chromosomal distribution of *ZmPLC* genes **(A)** and synteny analysis between *ZmPLCs* and *PLCs* from *Arabidopsis* or rice **(B)**. The number of the chromosome is shown on each chromosome. Gray lines in the background indicate the collinear blocks within maize and other plant genomes, while the red lines highlight the syntenic *PLC* gene pairs.

According to the data of the gene locus, 11 *ZmPLC* genes were found in five different chromosomes (Table 1, Figure 3A). *ZmPI-PLC2, ZmNPC1a, ZmNPC3*, and *ZmNPC4* are localized on chromosome 1, *ZmPI-PLC1* and *ZmPI-PLC4* are distributed on chromosomes 2 or 9, and *ZmNPC2* and *ZmNPC5* are located on chromosomes 3, while chromosomes 5 contained *ZmNPC1b, ZmPI-PLC3a*, and *ZmPI-PLC3b* (Figure 3A).

To understand more about the phylogeny of *ZmPLC* genes family, syntenic analysis was performed between maize and two other plant species, including *Arabidopsis thaliana* and *Oryza sativa* (Figure 3B). There were three *ZmPLC* genes that were synchronized with those in *Arabidopsis thaliana* (Figure 3B, Supplementary Table 2). The comparative syntenic maps of maize associated with rice were analyzed further, and six out of 11 *ZmPLC* genes had collinear genes in rice (Figure 3B, Supplementary Table 2), indicating that these genes may be derived from a common ancestor.

### Analysis of *cis-*Elements in the Promoters of *ZmPLCs*

**Figure 4:**
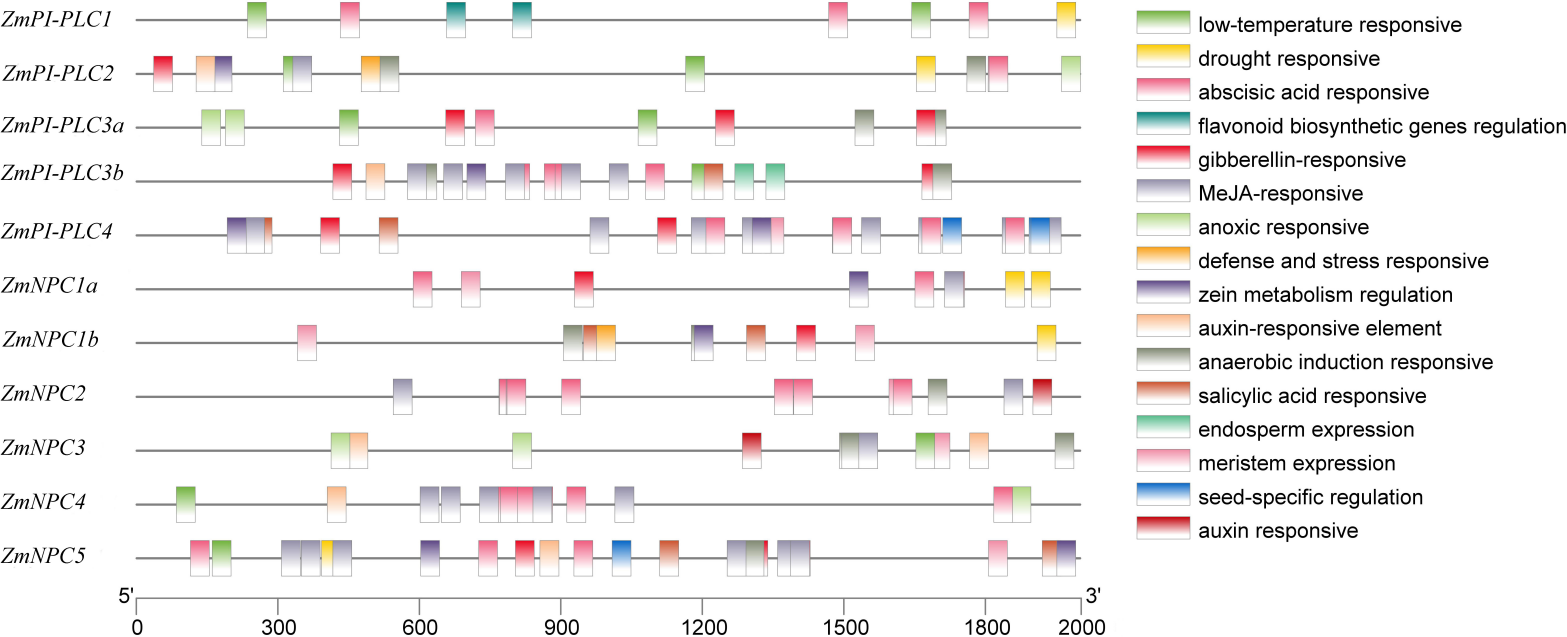
Putative regulatory *cis*-elements in *ZmPLC* family gene promoters. Different *cis*-elements are represented with different color box.

To study the expression regulation patterns and the potential function of *ZmPLCs*, putative *cis*-elements on promoter regions were searched in the Plant CARE database. *Cis*-elements related to developmental processes, such as the meristem expression (CAT-box), were found in the promoter regions of *ZmPI-PLC4, ZmNPC1a, ZmNPC1b, ZmNPC2, ZmNPC3*, and *ZmNPC5*, suggesting that *ZmPLC* genes play important roles in differentiation (Figure 4). The hormone-responsive elements, including abscisic acid (ABA) responsive elements (ABREs), MeJA-responsive elements (TGACG-motif and CGTCA-motif), salicylic acid-responsive element (TCA-element and SARE), auxin-responsive element (TGA-element and AuxRR-core), and gibberellin-responsive element (TATC-box, P-box, and GARE-motif), were also found in some *ZmPLC* gene promoters (Figure 4), showing that *ZmPLC* genes may play a role in growth and development. Furthermore, stress-responsive elements, such as low-temperature responsive (LTR) element, drought-responsive element, anaerobic induction responsive element (ARE), and defense and stress responsive element (AT-rich and TC-rich repeats), were observed in some promoters of *ZmPLC* genes. For example, LTR elements were detected in *ZmPI-PLC1/2/3a/3b* and *ZmNPC3/4/5* gene promoters (Figure 4), while drought-responsive elements were found in the promoters of *ZmPI-PLC1*/*2* and *ZmNPC1a/1b/5* (Figure 4).

### Expression Profiles of *ZmPLCs* in Different Tissues and Developmental Stages of Maize

**Figure 5:**
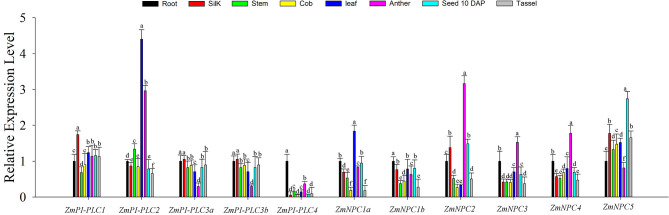
Tissue-specific expression patterns of the 11 *ZmPLC* genes in maize root, stem, leaf, silk, immature cob, anther, and kernel [10 days after pollination (DAP)] by using qRT-PCR. The expression levels were normalized to that of TUB. Bars represent SE (*n* = 3). The entire experiment was repeated three times. Different letters indicate values that departed significantly from those of root.

To study the roles of *ZmPLCs* in growth and development of maize, qRT-PCR was used to determine the expression profiles of the 11 *ZmPLC* mRNAs in different tissues, including root, stem, leaf, silk, immature cob, anther, tassel, kernel (10 DAP) (Figure 5). The results showed that *ZmPLCs* were significantly different under various different tissues. For example, *ZmPI-PLC1* was more highly expressed in silk; *ZmPI-PLC2* and *ZmNPC1a* were more highly expressed in the leaf than in other tissues; *ZmPI-PLC3a* and *ZmPI-PLC3b* had similar expression patterns with relatively lower expressions in anther, while *ZmNPC2, ZmNPC3*, and *ZmNPC4* had relatively high expressions in anther (Figure 5). Except for the relatively high expression in root, *ZmPI-PLC4* and *ZmNPC1b* showed low expression in others organs (Figure 5), and *ZmNPC5* was relatively highly expressed in 10 DAP of kernel (Figure 5). These results indicated that *ZmPLCs* may have important functions in different developmental stages and tissues of maize.

### Expression Profiling of *ZmPLCs* in Response to Various Stresses

**Figure 6:**
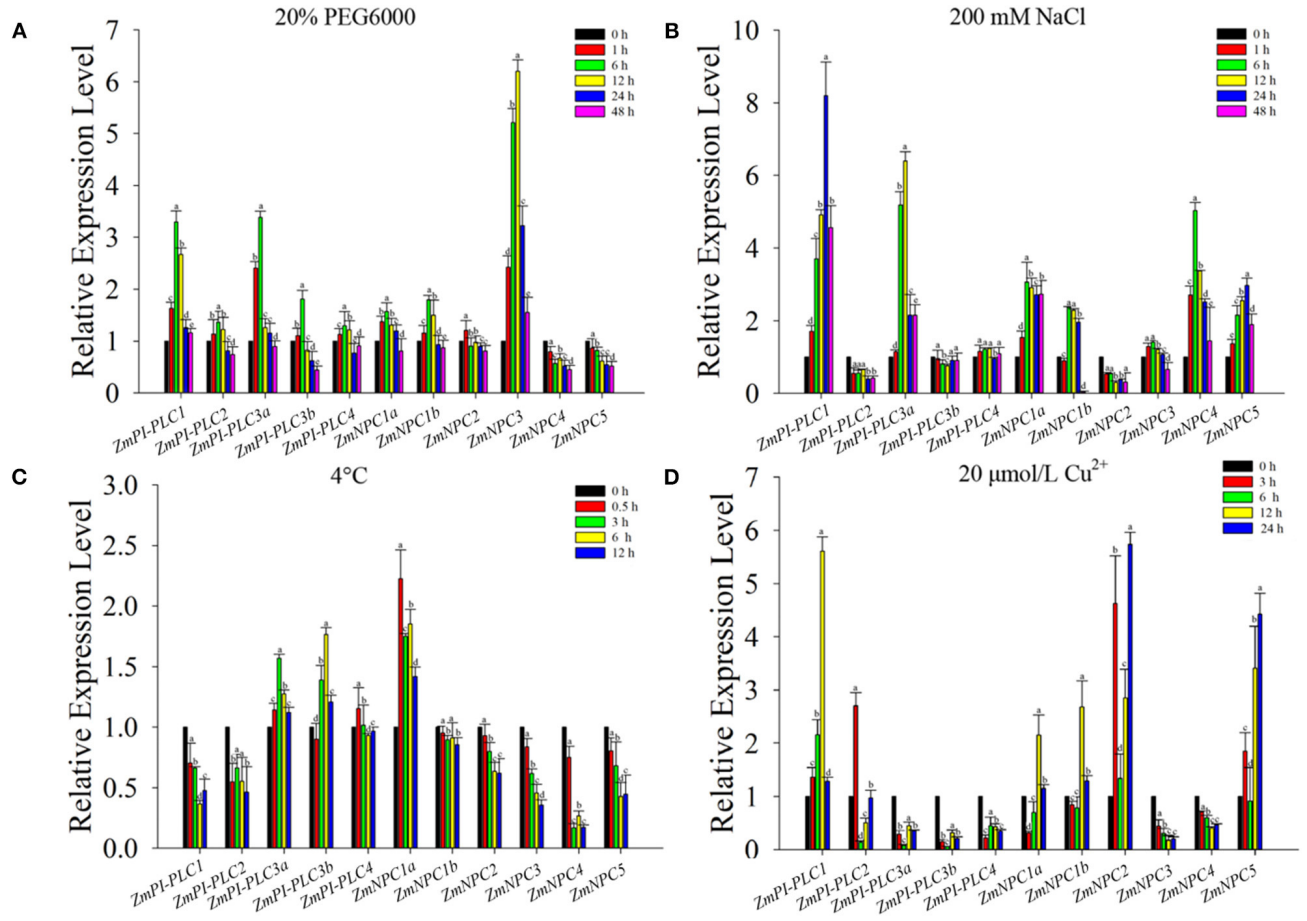
Quantitative real-time PCR analysis of *ZmPLCs* expression under stress treatments: **(A)** 20% PEG 6000, **(B)** 200 mM NaCl, **(C)** 4°C, **(D)** 20 μmol/L Cu^2+^. The expression levels were normalized to that of TUB. Bars represent SE (*n* = 3). The entire experiment was repeated three times. Different letters indicate values that departed significantly from those of without stress.

To verify the expression changes of *ZmPLCs* under stresses, the expression profiles were examined by qRT-PCR under different abiotic stress treatments, including 20% PEG6000, 200 mM NaCl, 4°C, and 20 μmol/L Cu^2+^ (Figure 6). The results show that *ZmPLC* genes, except *ZmNPC4* and *ZmNPC5*, were induced by 20% PEG 6000, peaking at 6 or 12 h after treatments, and then reduced to the lowest level at 48 h (Figure 6A). *ZmPI-PLC1, ZmPI-PLC3a, ZmNPC1a, ZmNPC1b, ZmNPC4*, and *ZmNPC5* were rapidly induced by high salinity, and *ZmPI-PLC3b, ZmPI-PLC4*, and *ZmNPC3* mRNAs were almost unaffected; however, the expressions of *ZmPI-PLC2* and *ZmNPC2* were suppressed (Figure 6B). The expressions of *ZmPI-PLC1, ZmPI-PLC2, ZmNPC1b, ZmNPC2, ZmNPC3, ZmNPC4*, and *ZmNPC5* were decreased after cold treatment and then reached the lower level at 12 h, while *ZmPI-PLC3a, ZmPI-PLC3b*, and *ZmNPC1a* mRNAs were induced with cold treatment, then still relatively high level at 12 h (Figure 6C). Under 20-μmol/L Cu^2+^ treatments, *ZmPI-PLC3a, ZmPI-PLC3b, ZmPI-PLC4, ZmNPC2*, and *ZmNPC3* mRNAs were suppressed within 48 h, while *ZmPI-PLC1, ZmPI-PLC2, ZmNPC1a, ZmNPC1b, ZmNPC2*, and *ZmNPC5* were induced within 48 h and peaked at 3 or 6 h after treatment (Figure 6D). These results show that *ZmPLCs* genes might play important roles in response to various abiotic stresses.

### Subcellular Localization of ZmPLC Proteins

**Figure 7:**
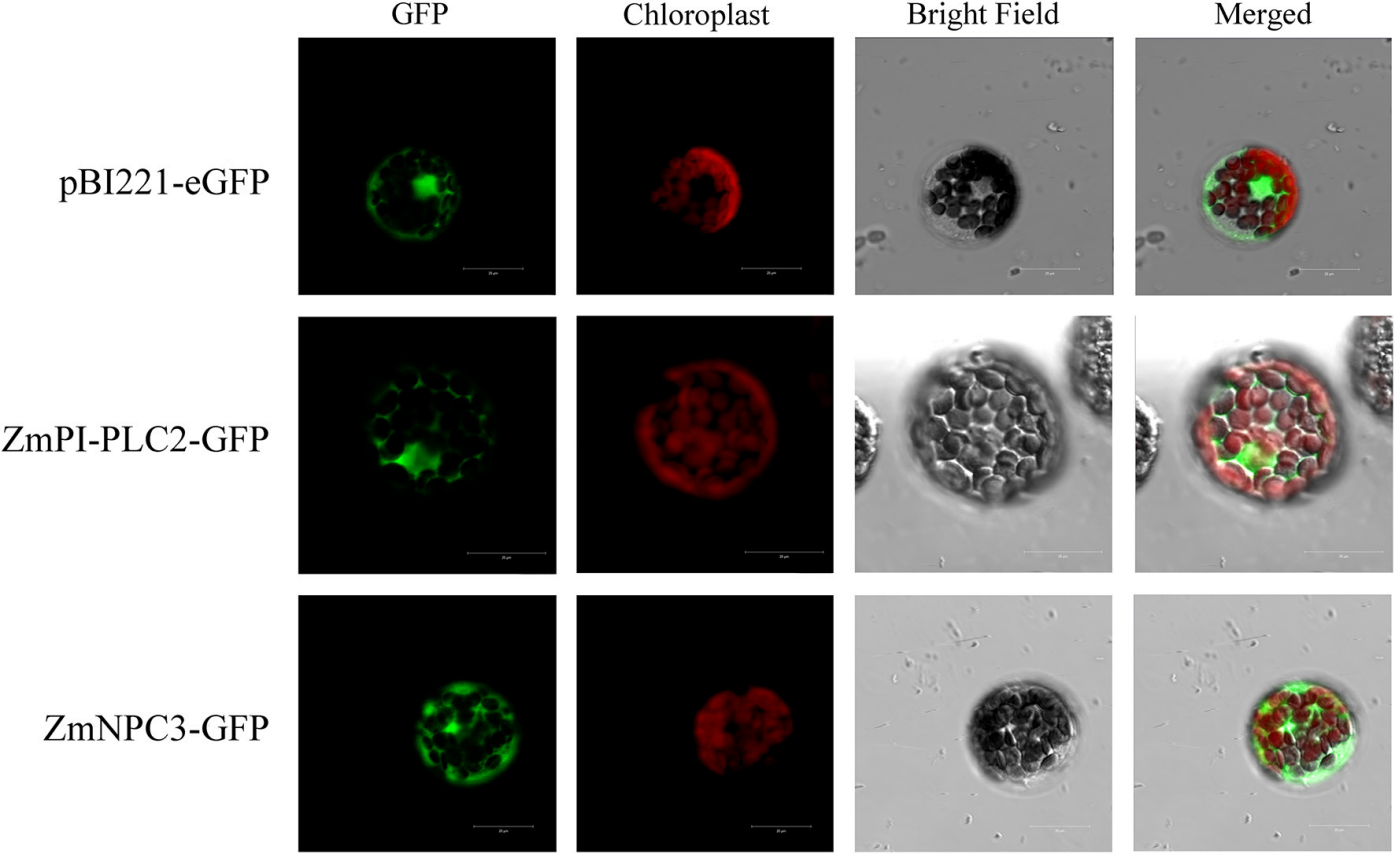
Subcellular localization of ZmPLC proteins in *Arabidopsis* leaf protoplasts.

Previously, it was reported that the substrates of PLCs, PI4P and PI(4,5)P_2_, are located in the plasma membrane (Munnik et al., 1998), so it is speculated that the subcellular localization of PLCs should also be in the plasma membrane; however, the sub-localization prediction shows that ZmPLCs may be multi-localized (Table 1). In order to confirm the subcellular localization, two randomly selected ZmPLCs, ZmPI-PLC2 and ZmNPC3, were transiently expressed in *A. thaliana* mesophyll protoplasts to analyze subcellular localization by fusing to the N-terminus of GFP. In *Arabidopsis* leaf protoplasts, the fluorescence of the vector control was distributed throughout the nucleus and cytoplasm (Figure 7); however, the signals of ZmPI-PLC2 and ZmNPC3 were only observed in the cytoplasm (Figure 7), showing that ZmPI-PLC2 and ZmNPC3 are located in the cytosol.

### Regional Association Mapping of *ZmPLCs* for Agronomic Traits in Maize

**Figure 8:**
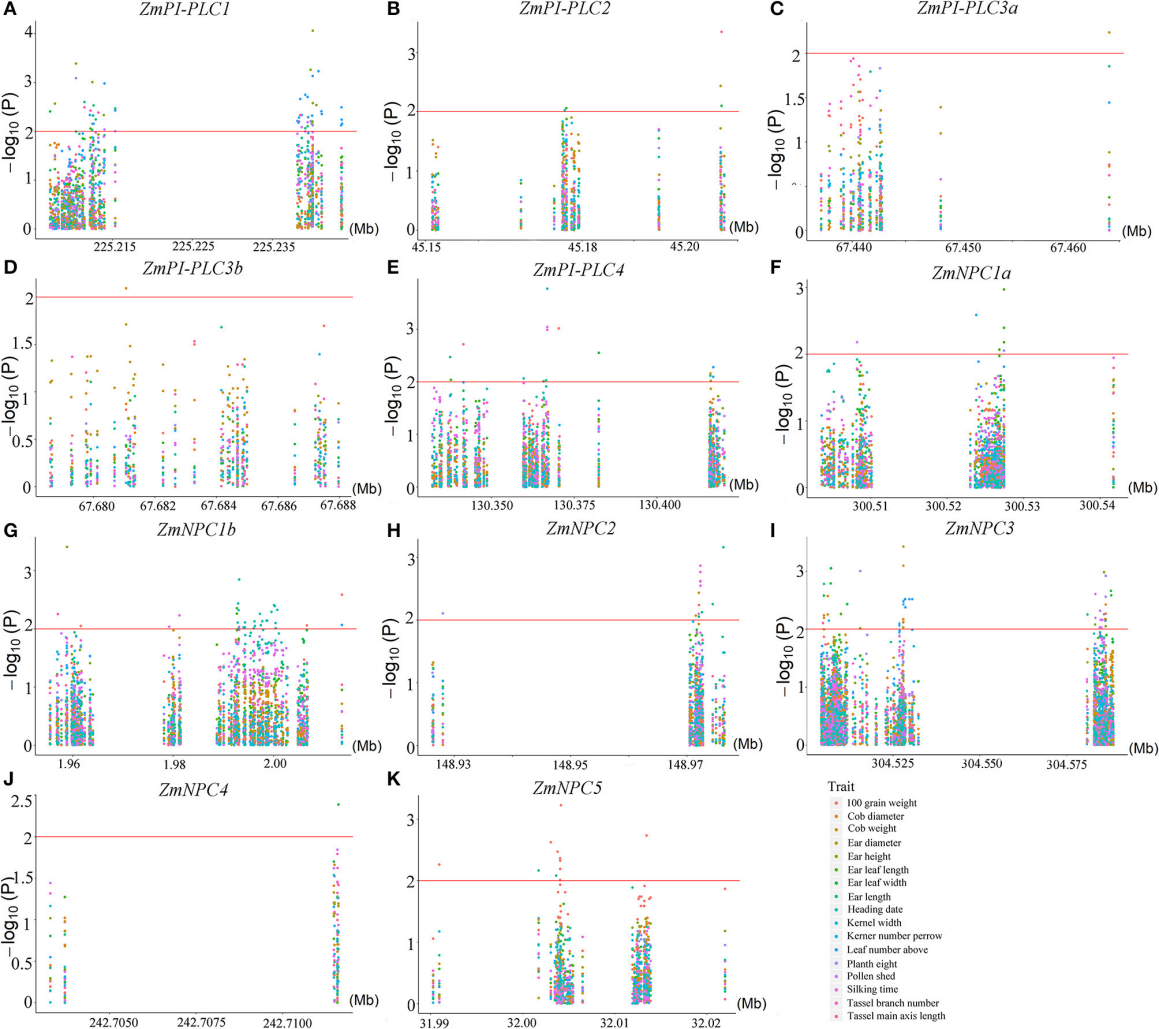
Regional correlation analysis between *ZmPLC* genes with the agronomic traits in maize. **(A–K)** show the correlation between *ZmPLC* genes with the agronomic traits, respectively. Local Manhattan plot (top) and linkage disequilibrium heatmap (bottom) surrounding the SNPs for agronomic traits on the range of 100 kb upstream/downstream of *ZmPLC* genes. An logarithm of the odds (LOD) > 2 indicates that a SNP is significantly related to kernel-related traits.

To understand the possible functions of the *ZmPLCs*, an analysis of agronomy -related trait QTLs from 508 maize inbred lines was performed. There were significant correlations between all 11 *ZmPLC* genes and more than one agronomic trait at the P ≤ 0.05 level, such as kerner number per row, 100 grain weight, cob weight, and so on (Figure 8). At P ≤ 0.01, 10 *ZmPLC* genes were identified to be related to some important agronomic traits (Supplementary Table 3). For example, *ZmPI-PLC1* was significantly correlated with ear height, ear leaf length, ear length, kerner number per row, plant height, pollen shed, and tassel branch number (Supplementary Table 3, Figure 8A); *ZmPI-PLC4* significantly affected heading date, pollen shed, 100 grain weight, silking time, ear leaf width, cob weight, kerner number per row, and so on (Supplementary Table 3, Figure 8E); *ZmNPC1a* significantly affected ear leaf length, kerner number per row, pollen shed, and plant height (Supplementary Table 3, Figure 8F).

## Discussion

### *ZmPLC* Gene Identification and Evolutionary Analysis in Maize

At present, *PLC* genes and their functions in many plants have been studied and reported, such as *Arabidopsis*, rice, and soybean (Wang et al., 2015; Zhang et al., 2018). It was reported that nine, 15, and 19 *PLC* genes were found in rice (Singh et al., 2013), *Arabidopsis* (Zheng et al., 2012), and cotton (Zhang et al., 2018), respectively. In this study, 11 ZmPLCs, five PI-PLCs, and six NPC members were identified according to the domain structure and phylogenetic analysis (Supplementary Figure 1, Figure 2). Based on the phylogenetic analysis, although PLCs from closely related species made a phylogenetic clade due to different genetic relationships (Figure 1), maize PI-PLCs made a phylogenetic clade with dicots PI-PLCs with very low bootstrap; however, maize NPCs fall in the same clade with dicot NPCs with a relative high bootstrap (Figure 1), suggesting that PLCs from different species diversify during the course of evolution even after being originated from a common ancestor. In addition, there are generally more PLCs in dicots than monocots (Zheng et al., 2012; Zhang et al., 2018), especially PI-PLC, which indicates that the natural selection of the *PLC* genes is a variant for different plant species. Domain and motif analysis of the PI-PLC protein sequences in maize with homologs from other plants contained the conserved and typical PI-PLC-X and PI-PLC-Y catalytic domains and C2 domain (Supplementary Figure 1B, Figure 2). It is worth noting that an EF hand-like motif was only found in ZmPI-PLC4 (Supplementary Figure 1A). Similarly, all the NPC members harbored a highly conserved phosphoesterase domain (Supplementary Figure 1A). The gene structure of *PLCs* from different species could be distinguished into two types: exon-rich (PI-PLC) and exon-poor (NPC) (Figure 2). Genes in the same branch usually have similar exon–intron structures, whereas the gene structures differed markedly among the different branches (Figure 2). Furthermore, there are similar results in the motif compositions of the PLC protein (Figure 2). Synteny analysis showed that the number of *PLC* homologous genes between maize and rice were more than those between maize and *Arabidopsis*, which indicating that PLC has a higher homology among related species (Figure 3). These findings indicated that highly conserved PLC from different plants might have a similar function in the evolution of plants.

### Functional Analysis of the *ZmPLCs* Response to the Development and Abiotic Stress in Maize

The *cis*-regulatory elements in the promoter play an important role in regulating the expression patterns of the genes (Vedel and Scotti, 2011). According to analysis of the *cis*-acting elements in *ZmPLC* promoter region, the *ZmPLC* promoters contain various element responses to plant development and stresses, for example, light-responsive elements, ABA-response elements, MeJA-responsive elements, LTR element, and so on (Figure 4). These results mean that *ZmPLC* genes could be involved not only in the regulation of the growth and development of maize but also in various stress responses.

The expression patterns of *PLC* genes have already been studied in different tissues and developments of many plants, such as *Arabidopsis*, rice, soybean, and so on. In this study, the expression profiles of *ZmPLC* genes were validated using qRT-PCR under different tissues. All *ZmPLC* genes were expressed in our tested tissues, while the expression abundance of different *ZmPLC* genes is significantly different in tissues (Figure 5). For example, the transcript levels of *ZmPI-PLC2* were relatively high in all organs, especially leaf and anther (Figure 5). Interestingly, *AtPI-PLC2*, involved in reproductive organ development, was also highly expressed in leaves, stems, roots, and flowers (Tasma et al., 2008; Li et al., 2015). *OsPLC* genes from rice are also expressed differentially during reproductive developmental phases including stages of panicle and seed development (Singh et al., 2013). Furthermore, the regional association analysis between 11 *ZmPLCs* and 17 agronomic traits in maize indicated that some *ZmPLC* genes are significantly correlated with many development-related traits (Figure 8). These results suggested that *ZmPLC* genes have important functions in the regulation of growth and development in maize and could act as important candidate genes to improve maize agronomic traits for breeding.

Many previous studies have reported that *PLC* members are involved in various abiotic stress-triggered signaling transductions in many plant species. For example, *AtPI-PLC3, AtPI-PLC9*, and *AtNPC1* genes play an important function in regulating heat tolerance (Gao et al., 2014; Krčková et al., 2015), whereas *AtNPC4* and *AtNPC5* genes could respond to salt stress (Kocourková et al., 2011; Peters et al., 2014). *OsPI-PLC4* plays a positive role in osmotic stress response (Deng et al., 2019). Overexpression of maize *PI-PLC1* could enhance drought tolerance of transgenic plants (Wang et al., 2008). In this study, the expression of *ZmPLC* genes showed different changes under different stresses; however, they presented their own characteristics (Figure 6). For example, all maize *PI-PLC* genes were induced by osmotic treatments, while only *ZmPI-PLC1* and *ZmPI-PLC3a* were rapidly induced by high salinity (Figures 6A,B). *ZmPI-PLC3a* and *ZmPI-PLC3b* were upregulated by cold treatment and suppressed by copper ion treatment (Figures 6C,D). *ZmNPC4* and *ZmNPC5* were downregulated by osmotic treatments; however, they were rapidly induced by high salinity (Figure 6B). The different expression changes of *ZmPLCs* under different stresses may be related to the *cis*-acting elements in their promoters; for example, the *cis*-regulatory elements including MBS (MYB binding site), ABRE, and defense and stress responsive element (AT-rich and TC-rich repeats) are known to regulate various stress responses (Abe et al., 2003; Narusaka et al., 2003). In this study, the LTR could be detected in *ZmPI-PLC1/2/3a/3b* and *ZmNPC3/4/5* gene promoters (Figure 4), and *ZmPI-LC3a* and *ZmPI-PLC3b* mRNAs were induced by cold treatments.

The substrates of PLCs, phosphoinositides, PC, or PE, are mainly distributed in the plasma membrane (Munnik et al., 1998), so it is speculated that the subcellular localization of PLCs should also be in the plasma membrane. However, the sub-localization from many plants showed that PLCs maybe multi-localized. For example, *Arabidopsis* AtPLC9 and soybean GmPLC10-GFP fluorescence were located in the plasma membrane (Zheng et al., 2012), OsPLC1 and OsPLC4 were distributed throughout the cytoplasm and nucleus, while OsNPC3 protein might be localized in the chloroplast/plastids (Singh et al., 2013). DAG derived from PI-PLC or NPC activities can be phosphorylated to PA by DGK, while DAG could act as a substrate to produce various lipid species and also significantly affect properties of cell membranes as sites of crucial cell activity (Darwish et al., 2009; Hong et al., 2016). On the other hand, PI-PLC hydrolyzes PtdIns(4,5)P_2_ into Ins(1,4,5)P_3_, and Ins(1,4,5)P_3_ is synthesized into InsP_6_, which could bind to IP_6_ receptors and lead to release of Ca^2+^ (Hong et al., 2016). Ca^2+^ and PA could act as a signaling molecule to play vital roles in various signaling pathways such as plant development, hormone signaling, and abiotic or biotic stresses to produce favorable response to plants (Xue et al., 2007). There is a possibility that the diverse localization of plant PLCs implies that they might take part in different cellular processes during development and abiotic stress.

## Conclusions

In this study, 11 *ZmPLCs* were identified by genome-wide analysis. The results of evolution, gene structures, and motifs of PLCs indicated that ZmPLCs were highly conserved compared with their homologous genes from other plants. Additionally, the *cis*-elements, expression profiles, and association analysis between *ZmPLC* genes and agronomic traits for *ZmPLC* genes were also analyzed, which showed that *ZmPLC* genes may have important functions in regulating development and various stresses. Taken together, these results provide useful information for further study of the roles of *ZmPLCs* in plant development and environmental stress conditions.

## Data Availability Statement

The datasets presented in this study can be found in online repositories. The names of the repository/repositories and accession number(s) can be found in the article/Supplementary Material.

## Author Contributions

JZ and HL wrote the paper. JZ, YZ, and JL performed the experiments. JZ contributed to the data analysis. All authors read and approved the manuscript.

## Conflict of Interest

The authors declare that the research was conducted in the absence of any commercial or financial relationships that could be construed as a potential conflict of interest.

## Supplementary Material

Supplementary File 1 List of the PLC sequences from multi-species in this study.

Supplementary Figure 1 Schematic domain structures of ZmPLC **(A)** and Multiple sequence alignments of ZmPLCs **(B)**. Protein sequences of ZmPLC were aligned by Bioedit. The conserved and characteristic domains and sequences of PI-PLCs and NPCs have been marked with colored box and red underline, respectively.

Supplementary Table 1 Primers and their sequences used in this study.

Supplementary Table 2 Synteny blocks of *PLC* genes between maize and two representative plant species.

Supplementary Table 3 The *ZmPLC* genes list associated with agronomic traits in maize. The red mark indicates the significant value after Bonferroni correction (*p* < 0.05/n).
