# Supplementary material for: Genome-Wide Investigation of the Phospholipase C Gene Family in Zea mays
Source: Front Genet. 2021 Jan 12;11:611414. doi: 10.3389/fgene.2020.611414 (PMC7835795; doi:10.3389/fgene.2020.611414)
Supplement: Supplementary File 1 — List of the PLC sequences from multi-species in this study. [file Table_2.DOCX]

>AtPI-PLC1

MKESFKVCFCCVRNFKVKSSEPPEEIKNLFHDYSQDDRMSADEMLRFVIQVQGETHADINYVKDIFHRLKHHGVFHPRGIHLEGFYRYLLSDFNSPLPLTREVWQDMNQPLSHYFLYTGHNSYLTGNQLNSNSSIEPIVKALRNGVRVIELDLWPNSSGKEAEVRHGGTLTSREDLQKCLNVVKENAFQVSAYPVVLTLEDHLTPILQKKVAKMVSKTFGGSLFQCTDETTECFPSPESLKNKILISTKPPKEYLQTQISKGSTTDESTRAKKISDAEEQVQEEDEESVAIEYRDLISIHAGNRKGGLKNCLNGDPNRVIRLSMSEQWLETLAKTRGPDLVKFTQRNLLRIFPKTTRFDSSNYDPLVGWIHGAQMVAFNMQSHGRYLWMMQGMFKANGGCGYVKKPDVLLSNGPEGEIFDPCSQNLPIKTTLKVKIYTGEGWNMDFPLDHFDRYSPPDFYAKVGIAGVPLDTASYRTEIDKDEWFPIWDKEFEFPLRVPELSLLCITVKDYDSNTQNDFAGQTCFPLSEVRPGIRAVRLHDRAGEVYKHVRLLMRFVLEPR

>AtPI-PLC2

MSKQTYKVCFCFRRRFRYTASEAPREIKTIFEKYSENGVMTVDHLHRFLIDVQKQDKATREDAQSIINSASSLLHRNGLHLDAFFKYLFGDNNPPLALHKVHHDMDAPISHYFIFTGHNSYLTGNQLSSDCSEVPIIDALKKGVRVIELDIWPNSNKDDIDVLHGMTLTTPVGLIKCLKAIRAHAFDVSDYPVVVTLEDHLTPDLQSKVAEMVTEIFGEILFTPPVGESLKEFPSPNSLKRRIIISTKPPKEYKEGKDVEVVQKGKDLGDEEVWGREVPSFIQRNKSEAKDDLDGNDDDDDDDDEDKSKINAPPQYKHLIAIHAGKPKGGITECLKVDPDKVRRLSLSEEQLEKAAEKYAKQIVRFTQHNLLRIYPKGTRVTSSNYNPLVGWSHGAQMVAFNMQGYGRSLWLMQGMFRANGGCGYIKKPDLLLKSGSDSDIFDPKATLPVKTTLRVTVYMGEGWYFDFRHTHFDQYSPPDFYTRVGIAGVPGDTVMKKTKTLEDNWIPAWDEVFEFPLTVPELALLRLEVHEYDMSEKDDFGGQTCLPVWELSEGIRAFPLHSRKGEKYKSVKLLVKVEFV

>AtPI-PLC3

MSESFKVCFCCSRSFKEKTRQPPVSIKRLFEAYSRNGKMSFDELLRFVSEVQGERHAGLDYVQDIFHSVKHHNVFHHHGLVHLNAFYRYLFSDTNSPLPMSGQVHHDMKAPLSHYFVYTGHNSYLTGNQVNSRSSVEPIVQALRKGVKVIELDLWPNPSGNAAEVRHGRTLTSHEDLQKCLTAIKDNAFHVSDYPVIITLEDHLPPKLQAQVAKMLTKTYRGMLFRRVSESFKHFPSPEELKGKILISTKPPKEYLESKTVHTTRTPTVKETSWNRVANKILEEYKDMESEAVGYRDLIAIHAANCKDPSKDCLSDDPEKPIRVSMDEQWLDTMVRTRGTDLVRFTQRNLVRIYPKGTRVDSSNYDPHVGWTHGAQMVAFNMQGHGKQLWIMQGMFRGNGGCGYVKKPRILLDEHTLFDPCKRFPIKTTLKVKIYTGEGWDLDFHHTHFDQYSPPDFFVKIGIAGVPRDTVSYRTETAVDQWFPIWGNDEFLFQLSVPELALLWFKVQDYDNDTQNDFAGQTCLPLPELKSGVRAVRLHDRTGKAYKNTRLLVSFALDPPYTFR

>AtPI-PLC4

MEGKKEMGSYKFCLIFTRKFRMTESGPVEDVRDLFEKYTEGDAHMSPEQLQKLMTEEGGEGETSLEEAERIVDEVLRRKHHIAKFTRRNLTLDDFNYYLFSTDLNPPIADQVHQNMDAPLSHYFIFTGHNSYLTGNQLSSNCSELPIADALRRGVRVVELDLWPRGTDDVCVKHGRTLTKEVKLGKCLESIKANAFAISKYPVIITLEDHLTPKLQFKVAKMITQTFGDMLYYHDSQGCQEFPSPEELKEKILISTKPPKEYLEANDTKEKDNGEKGKDSDEDVWGKEPEDLISTQSDLDKVTSSVNDLNQDDEERGSCESDTSCQLQAPEYKRLIAIHAGKPKGGLRMALKVDPNKIRRLSLSEQLLEKAVASYGADVIRFTQKNFLRIYPKGTRFNSSNYKPQIGWMSGAQMIAFNMQGYGRALWLMEGMFRANGGCGYVKKPDFLMDASPNGQDFYPKDNSSPKKTLKVKVCMGDGWLLDFKKTHFDSYSPPDFFVRVGIAGAPVDEVMEKTKIEYDTWTPIWNKEFTFPLAVPELALLRVEVHEHDVNEKDDFGGQTCLPVSEIRQGIRAVPLFNRKGVKYSSTRLLMRFEFV

>AtPI-PLC5

MKRDMGSYKMGLCCSDKLRMNRGAPPQDVVTAFVEYTEGRSHMTAEQLCRFLVEVQDETEVLVSDAEKIIERITCERHHITKFLRHTLNLDDFFSFLFSDDLNHPIDSKVHQDMASPLSHYFIYTSHNSYLTGNQINSECSDVPLIKALKRGVRALELDMWPNSTKDDILVLHGWAWTPPVELVKCLRSIKEHAFYASAYPVILTLEDHLTPDLQAKAAEMMKEIFMDMVYFPEAGGLKEFPSPEDLKYKIVISTKPPKGSLRKDKDSESDASGKASSDVSADDEKTEEETSEAKNEEDGFDQESSNLDFLTYSRLITIPSGNAKNGLKEALTIDNGGVRRLSLREQKFKKATEMYGTEVIKFTQKNLLRIYPKATRVNSSNYRPYNGWMYGAQMVAFNMQGYGRALWMMHGMFRGNGGCGYVKKPDFMMNNNLSGEVFNPKAKLPIKKTLKVKVYMGKGWDSGFQRTCFNTWSSPNFYTRVGITGVRGDKVMKKTKKEQKTWEPFWNEEFEFQLTVPELALLRIEVHDYNMPEKDDFSGQTCLPVSELRQGIRSVPLYDRKGERLVSVTLLMRFHFL

>AtPI-PLC6

MGKEKKTESYNNDSGSYNYRMFKFYNRKFKINEVTPTDDVRDAFCQFAVGGGGGGTDGDSSDGDGSTGVMGAEQLCSFLDDHGESTTVAEAQRLIDEVIRRRHHVTRFTRHGLDLDDFFNFLFYDDLNPPITPHVHQDMTAPLSHYFIYTGHNSYLTGNQLSSDCSEVPVIKALQRGVRVIELDLWPNSTGTDINVLHGRTLTTPVPLMKCLKSIRDYAFSSSPYPVIITLEDHLTPDLQAKVAEMATQIFGQMLYYPESDSLLEFPSPASLLHRIIISTKPPKEYLESRNPIVKQKDNNVSPSSEDETPRTEEIQTLESMLFDQDFESKSDSDQEDEEASEDQKPAYKRLITIHAGKPKGTVKEEMKVVVDKVRRLSLSEQELDRTCSSNSQDVVRFTQRNLLRIYPKGTRFNSSNYKPLIGWTHGAQMIAFNMQGYGKSLWLMHGMFRANGGCGYVKKPNFLMKKGFHDEVFDPRKKLPVKETLKVK

>AtPI-PLC7

MSKQTYKVCFCFRRRYRHTVSVAPAEIKTLFDNYSDKGLMTTDLLLRFLIDVQKQDKATKEEAQDIVNASSSLLHRNGLHLDAFFKYLFAVTNSPLSSLEVHQDMDAPLSHYFIYTGHNSYLTGNQLSSDCSELPIIEALKKGVRVIELDIWPNSDEDGIDVLHGRTLTSPVELIKCLRAIREHAFDVSDYPVVVTLEDHLTPKLQAKVAEMVTDIFGEMLFTPPSGECLKEFPSPAFLKKRIMISTKPPKEYKAATDDDLVKKGRDLGDKEVWGREVPSFIRRDRSVDKNDSNGDDDDDDDDDDDDDDGDDKIKKNAPPEYKHLIAIEAGKPKGGITECLKVDPDKVRRLSLSEEQLEKASEKYAKQIVRFTQRNLLRVYPKGTRITSSNYNPLIAWSHGAQMVAFNMQGLGRSLWVMQGMFRGNGGCGYIKKPDLLLKSNAVFDPEATLPVKTTLRVTIYMGEGWYYDFPHTHFDRYSPPDFYTRVGIAGVPADTVMKKTKTLEDNWIPAWDEVFEFPLTVPELALLRIEVHEYDMSEKDDFGGQICLPVWELRQGIRAVPLRNQDGVKCRSVKLLVRLEFVVYMGDGWRMDFSHTHFDAYSPPDFYTKMFIVGVPADNAKKKTKIIEDNWYPIWDEEFSFPLTVPELALLRIEVREYDMSEKDDFGGQTCLPVAELRPGIRSVPLYDKKGEKMKSVRLLMRFIFE

>AtPI-PLC8

MLVTRRWESHPANSPDLILQFFGNEFHGYGDDMPETLRRLTELLGYEKEEDGAGMNAAKKIAAELNRRKDDIPAFRRLRCLELDQLNEFLFSTKLNPPIGDQVHHDMHAPLSHYFIHTSLNSYFTGNVFGKYSILPIIEALEQGVRVVELDLWPDGRGSICVRPSWNFEKPLKLQECLDSIKEHAFTKCTYPLIITFKDGLKPELQSKATQMIQQTFNHMVYHHDPHSLEVFPSPQQLRNKILISRRPPKELLYANDDDGKVGVRNGVEIRQHPADPNYQSLVSFHVVEPRGMLQNVLTGKANKIQRPGWYETDIISFTQKRFLRTRPQRKLLIYAPYKPQRAWMHGAQLIALSRKEEKEKLWLMQGMFRANGGCGYVKKPDFLLNAGPSGVFYPTVNPVVVKILKVKIYMGDGWIVDFKKRIGRLSKPDLYVRISIAGVPHDENIMKTTVKNNEWTPTWGEEFTFPLTYPDLALISFEVYDYEVSTADAFCGQTCLPVSELIEGIRAVPLYDERGKACSSTMLLTRFKWS

>AtPI-PLC9

MVNLRKKFEMKQANQPGRVPNYFRNKYHGYDDDMPNLLPTFIKLLDTEKDEDGAGLNAAEQIDRELKSRKCDILKFRNLTILELPHLNEFLFSTELNPPISDQVRHRDMNAPLSHYFIHTSLKSYFTGNNVFGRLYSIEPIIDALKQGVRVVELDLLPFGKDGICVRPKWNFEKPLELQECLDAIKQHAFTPTRSYPVIITIKDSLKPDLQSKVTQMIDQTFGDMVYHEDPQQSLEEFPSPAELQNKILISRRPPTKLLYAKAVENGVELEIQEGSTDKNYQSVVGFHAVEPRGMLQKALTDDVQQPGWYERDVISFTQNKFLRTRPKKRNLLSNPPYKPQRAWMHGAQMIALSRQDDKEKLWLMQGMFRANGGCGYVKKPNFLLNAGSSGVFYPTENPVVVKTLKVKIYMGDGWIVDFKKRIGRLSKPDLYVRISIAGVPHDEKIMNTTVKNNEWKPTWGEEFTFPLTYPDLALISFEVYDYEVSTPDYFCGQTCLPVSELIEGIRAVPLYDERGKACSSTMLLTRFKWS

>AtNPC1

MAFRRVLTTVILFCYLLISSQSIEFKNSQKPHKIQGPIKTIVVVVMENRSFDHILGWLKSTRPEIDGLTGKESNPLNVSDPNSKKIFVSDDAVFVDMDPGHSFQAIREQIFGSNDTSGDPKMNGFAQQSESMEPGMAKNVMSGFKPEVLPVYTELANEFGVFDRWFASVPTSTQPNRFYVHSATSHGCSSNVKKDLVKGFPQKTIFDSLDENGLSFGIYYQNIPATFFFKSLRRLKHLVKFHSYALKFKLDAKLGKLPNYSVVEQRYFDIDLFPANDDHPSHDVAAGQRFVKEVYETLRSSPQWKEMALLITYDEHGGFYDHVPTPVKGVPNPDGIIGPDPFYFGFDRLGVRVPTFLISPWIEKGTVIHEPEGPTPHSQFEHSSIPATVKKLFNLKSHFLTKRDAWAGTFEKYFRIRDSPRQDCPEKPEVKLSLRPWGAKEDSKLSEFQVELIQLASQLVGDHLLNSYPDIGKNMTVSEGNKYAEDAVQKFLEAGMAALEAGADENTIVTMRPSLTTRTSPSEGTNKYIGSY

>AtNPC2

MSIKAFALIQLLSVTILYNHVHATSPIKTIVVVVMENRSFDHMLGWMKKLNPEINGVDGSESNPVSVSDPSSRKIKFGSGSHYVDPDPGHSFQAIREQVFGSNDTSMDPPPMNGFVQQAYSEDPSGNMSASVMNGFEPDKVPVYKSLVSEFAVFDRWFASVPSSTQPNRMFVHSGTSAGATSNNPISLAKGYPQRTIFDNLDDEEFSFGIYYQNIPAVLFYQSLRKLKYVFKFHSYGNSFKDHAKQGKLPAYTVIEQRYMDTLLEPASDDHPSHDVYQGQKFIKEVYETLRASPQWNETLLIITYDEHGGYFDHVPTPVRNVPSPDGIVGPDPFLFQFNRLGIRVPTIAVSPWIEKGTVVHGPNGSPFPSSEYEHSSIPATVKKLFNLSSPFLTKRDEWAGTFENILQIRKEPRTDCPETLPEPVKIRMGEANEKALLTEFQQELVQLAAVLKGDNMLTTFPKEISKGMTVIEGKRYMEDAMKRFLEAGRMALSMGANKEELVHMKTSLTGRRP

>AtNPC3

MVEETSSGGGSSASPIKTIVVLVQENRSFDHMLGWFKELNPEIDGVSESEPRSNPLSTSDPNSAQIFFGKESQNIDPDPGHSFQAIYEQVFGKPFSDESPYPDPKMNGFVQNAEAITKGMSEKVVMQGFPPEKLPVFKELVQEFAVCDRWFSSLPSSTQPNRLYVHAATSNGAFSNDTNTLVRGFPQRTVFESLEESGFTFGIYYQSFPNCLFYRNMRKLKYVDNFHQYHLSFKRHCKEGKLPNYVVIEPRYFKILSAPANDDHPKNDVVEGQNLVKEIYEALRASPQWNEILFVVVYDEHGGYYDHVPTPVIGVPNPDGLVGPEPYNFKFDRLGVRVPALLISPWIEPGTVLHEPNGPEPTSQFEHSSIPATLKKIFNLKSFLTKRDEWAGTLDAVINRTSPRTDCPVTLPELPRARDIDIGTQEEDEDLTDFQIELIQAAAVLKGDHIKDIYPFKLADKMKVLDAARYVEEAFTRFHGESKKAKEEGRDEHEIVDLSKGSTRHSTPKSFVQKLFSCLICDN

>AtNPC4

MIETTKGGSGSYPIKTIVVLVQENRSFDHTLGWFKELNREIDGVTKSDPKSNTVSSSDTNSLRVVFGDQSQYVNPDPGHSIQDIYEQVFGKPWDSGKPDPNPGHPNMSGFAQNAERNKKGMSSAVMNGFKPNALPVYKELVQNFAICDRWFASVPASTQPNRLYVHSATSHGATSNDKKLLLEGFPQKTIFESLDEAGFSFGIYYQFPPSTLFYRNLRKLKYLTHFHQYGIQFKKDCKEGKLPNYVVVEQRWFDLLSTPANDDHPSHDVSEGQKLVKEVYEALRSSPQWNEILFIITYDEHGGFYDHVPTPVDGVPNPDGILGPPPYNFEFNRLGVRVPTFFISPWIEPGTVIHGPNGPYPRSQYEHSSIPATVKTIFKLKDFLSKRDSWAGTFESVITRDSPRQDCPETLSTPIKLRGTMAKENAQLSEFQEDLVIMAAGLKGDYKNEELIHKLCKETCVADASKYVTNAFEKFLEESRKARDRGCDENDIVYCVDDDDDHVVIPPQSHSEASNAAAQPKTQTSFFNKLFSCFIRHD

>AtNPC5

MAETKKGSESYPIKTIVVLVQENRSFDHTLGWFKELNREIDGVMKSDQKFNPGFSSDLNSHNVVFGDQSQYVDPNPGHSIRDIYEQVFGKPWDSGHPDPNPGPATMSGFAQNAERKMKGMSSAVMNGFKPDALPVYKELVQNFAICDRWFASVPGATQPNRLFIHSATSHGTTNNERKLLIEGFPQKTIFESLDEAGFTFGIYYQCFPTTLFYRNLRKLKYLTRFHDYGLQFKKDCKEGNLPNYVVVEQRWYDLLLNPANDDHPSHDVSEGQKLVKEVYEALRSSPQWNEILFIITYDEHGGFYDHVPTPLDGVPNPDGILGPPPYNFEFNRLGVRVPTFFISPWIEPGTVLHGSNGPYLMSQYEHSSIPATVKKIFKLKDFLTKRDSWAGTFESVITRNSPRQDCPETLSNPVKMRGTVAKENAELSDFQEELVIVAAGLKGDYKNEELLYKLCKKTCVSDASKYVTKAFDKFVEESKKARERGGDENDIVFCVDDDDDHNVVKPPPSQSEPSHATPWSN

>AtNPC6

MKPSSASRFSLTFSHFLTLYCLLTQTHVAQGSHQWQSPIKTVVVLVLENRSFDHLLGWMKNSVNPTINGVTGQECNPVPNSTQTICFTSDAEFVDPDPGHSFEAVEQQVFGSGPGQIPSMMGFVEQALSMPGNLSETVMKGFRPEAVPVYAELVKEFAVFDRWFSSIPGPTQPNRLFVYSATSHGSTSHVKKQLAQGYPQKTIFDSLHSNDIDFGIYFQNIPTTLFYRNLRQLKYIFNLHQYDLKFKKDAAKGKLPSLTVIEPRYFDLKGLPANDDHPSHDVANGQKLVKEVYEALRSSPQWNETLLVITYDEHGGFYDHVKTPYVGIPNPDGNTGPAPGFFKFDRLGVRVPTIMVSPWIQKGTVVSEAKGPTESSEYEHSSIPATIKKLFNLSSNFLTHRDAWAATFEDVVSHLTTPRTDCPMTLPEVAPMRATEPKEDAALSEFQGEVVQLAAVLNGDHFLSSFPEEIGKKMTVKQAHEYVKGATSRFIRASKEAMKLGADKSAIVDMRSSLTTRPHN

>OsPI-PLC1

MGTYKCCLIFKRRFRWNDAPPPDDVRALFANHSAGGGPHMAADGLRAYLQATGQDGDVDMERLVEQIRQLQGRGGRIPRVGRALPLLTVDDFHRFLFSHELNPPIRHGQGQVHHDMAAPLSHYFIYTGHNSYLTGNQLSSDCSDLPIIRALQRGVRVIELDMWPNSSKDDISILHGRTLTTPVSLLKCLLSIKQHAFEASPYPVIITLEDHLTPDLQDKAAKMVLEVFGDILYYPDKDHLKEFPSPQDLKGRVLLSTKPPREYLQAKDGNAATIKEDAKAAATDDAAWGKEVPDIHSQIHSATKHDQREDDDDTDEDEDDEEEEQKMQQHLAPQYKHLITIKAGKPKGTLLDALQSDPEKVRRLSLSEQQLAKLADHHGTEIVRFTQRNLLRIYPKGTRVTSSNYNPFLGWVHGAQMVAFNMQGYGRALWLMHGFYKANGGCGYVKKPDFLMQTDPEVFDPKKSLSPKKTLKVKVYMGDGWRMDFTQTHFDQYSPPDFYARVGIAGVPADSVMKRTRAIEDNWVPVWEEDFTFKLTVPEIALLRVEVHEYDMSEKDDFGGQTVLPVSDLIPGIRAVALHDRKGIKLNNVKLLMRFEFE

>OsPI-PLC2

MNAPLSHYFIYTGHNSYLTGNQLSSDCSDIPIIKALQIGVRVIELDMWPNSSKDDVDILHGRTLTAPVSLIKCLKSIKEYAFVASPYPVIITLEDHLTSDLQAKVAKMVLEVFGDTLYYPESKHLQEFPSPEALRGRVILSTKPPKEYLESKGGTMKDRDIEPQFSKGQNEEAVWGTEVPDIQDEMQTADKQHENDILYTQRDVEEDDEKKMCQHHPLEYKHLITIKAGKPKGAVVDALKGDPDKVRRLSLSEQELAKVAAHHGRNIVSFTHKNLLRIYPKGTRFNSSNYNPFLGWVHGAQMVAFNMQGYGRSLWLMHGFYKANGGCGYVKKPDFMMQTCPDGNVFDPKADLPVKKTLKVKVYMGEGWQSDFKQTYFDTYSPPDFYAKVGIAGVPSDSVMQKTKAVEDSWVPVWEEEFVFPLTVPEIALLRVEVHEYDVSEDDFGGQTALPVSELRPGIRTVPLFDHKGLKFKSVKLLMRFEFV

>OsPI-PLC3

MGSYAYKYCMCFTRKFRSPAADPPPDVRAAFLAAGGGDGGLRRFLAQAQGETPAEVDRILALLSGGGGGGGGGGIAARLVGPRPGPAPSLDDFFGFLFNADLNPPIATQVHQDMSAPFSHYYIYTGHNSYLTGNQLNSDSSDIPIIKALQRGVRVIELDMWPNSAKNNIDILHGGTLTAPVQIIKCLKSIKEYAFCASPYPLVITLEDHLTPDLQAKVAEMLVKTFGNLLYIPSSDPINEFPSPESLMKKIIISTKPPEEYKKFLKSKDNQNINGGLANLAEEGSLRRIDSNAEESDGKDELDDQDEDSSDEDDPKFQQETACEYRELITIHAGKPKGHLKDALKVDPDKVRRLSLSETQLAKATASHGADVIRFTQKNILRVYPKGTRINSSNYDPMNAWTHGAQMVAFNMQGHDKALRLMQGFFRANGGCGYVKKPDFLLRTGPNGEVFDPNASMPVKKTLKVKVYMGDGWRMDFSKTHFDTFSPPDFYTRVGIAGVRADCVMKKTRTIEDQWVPMWDEEFTFPLTVPELAVLRIEVHEYDMSEKHDFGGQTCLPVSELKQGIRAVPLHDRRGTRYKSVRLLMRFDFL

>OsPI-PLC4

MTTYRVCCFLRRFRAASNEPSEELGDVFQAYADGGGGVMGEEALRRFLREVQGEAAGGGDDELEATAREVMAFAAEQRLLRKGGAAAAGGGLTVEGFHRWLCSDANAALDPQKRVYQDMGLPLSHYFIYTGHNSYLTGNQLSSGCSEVPIVKALHDGVRVIELDLWPNAAKDAVEVLHGRTLTSPVGLMKCLEAIREYAFVASPYPVILTLEDHLTPDLQSKVAKMIKETFGDMLYVSETENMAEFPSPDELKGKIIVSTKPPKEYLQTKNDADADEAGVWGEEITDDKVAATAMTTEEKCAAAEEAVAAAAVDEEMQEAETDKKTQHGVDNEYRRLIAIPLTRRKHDMDQDLKVDPDMVTRLSLGEKAYEKAIVTHGAHIIRFTQRKLLRIFPRSTRITSSNYNPLMGWRYGVQMVAANMQGHGRKLWLTQGMFRANGGCGYVKKPDILMNNDPDKLFDPTSKLPVKTRLKVTVYMGDGWRFDFRKTHFDKCSPPDFYARVGIAGVEADTRMEQTKVKMDTWIPAWDHEFEFPLSVPELALLRVEVHESDNHQKDDFGGQTCLPVWELRRGIRSVRLCDHRGEPLRSVKLLMRFDFT

>OsNPC1

MAGGGGRERRGGGRLLVGVLLLTLVVSGHCLESTHHRGLKRRRRKHEIHSPIKTVVVVVMENRSFDHILGWLSRTRPDIDGLNGTQSNRLNASDPSSPEIFVTDEAGYVDSDPGHGFEDIREQIFGSADTSAVPAPMSGFAQNARGMGLGMPQNVMSGFKPESVPVYAALADEFAVFDRWFASVPTSTQPNRLYVHSATSHGLTFNARKDLIHGFPQKTIFDSLEENGLSFGIYYQNIPATLFYQSLRRLKHLVKFHQYSLKFKLHAKWGKLPNYAVIEQRYFDCEMFPANDDHPSHDVARGQRFVKEVYETLRASPQWNETALIITYDEHGGFYDHVPTPVVGVPQPDGIVGPDPYYFKFDRLGVRVPSFLISPWIEKRTVIHEPNGPQDSSQYEHSSIPATVKKLFNLHSNFLTKRDAWAGTFENYFKIRKTPRTDCPEKLPEVTKSLQPFGPKEDSSLSEFQVELIQLASQLNGDHVLNTYPDIGRTMTVGEANRYAEDAVARFLEAGRIALRAGANESALVTMRPALTSRASPSSDLSSEL

>OsNPC2

MAVRRRRPGPVAAAVLLLLAVATQAAASPIKTVVVVVMENRSFDHMLGWMKRLNPEIDGVTGGEWNPTNASDPSSGRVYFGEGAEYVDPDPGHSFQEIRQQIFGSDDASGPARMDGFVQQARSLGDNMTAAVMNGFSPDSVAVYRELVGEFAVFDRWFASVPSSTQPNRLFVHSATSGGATSNNPELLAKGYPQRTIFDNVHDAGLSFGVYYQDVPAVLFYRNLRKLKYLTKFHPFHGAFRDHAARGSLPNYAVVEQHYMDSKSHPANDDHPSHDVFQGQMLVKEVYETLRASPQWNQTLMVVTYDEHGGFYDHVPTPVTGVPSPDGIVGPPPYNFAFDRLGVRVPAIVISPWINKGTVVHGPNGSPTATSEYEHSSIPATVKKLFDLPQDFLTKRDAWAGTFESVVQGRTEPRTDCPEQLPMPMRIRLTEANEEAKLSEFQQELVQLASVLNGDHQLSSLQDTIRDRMNVREGIAYMRGAVKRFFETGMSAKRMGVDDEQIVKMRPSLTTRTSPAIEQP

>OsNPC3

MAGKIKTVVVLVQENRSFDHMLGWMKSLNPEIDGVTGAEFNRATAGDPSSPAIHFGDGSGYVDPDPGHSFQAIYEQVYGDAYTWGTTSPATKPGVPSPPMSGFAQEAEKERAGMSSTVMNGFRPEKVPVYRELVREFAVCDRWFASVPTSTQPNRMFVHSATSHGLVSNDGKQLRAGLPQRTIFDALHDAGHSFGVYYQFPPSVLFYRNMRQLKYVGNFHPYDTAFKRDCKAGKLPNYVVIEQRYFDLKLLPGNDDHPSHDVAHGQRLVKDVYEALRSSPQWHEILFVITYDEHGGFFDHVPTPVAGVPSPDGIVSAAPVSFAFDRLGVRVPTLLVSPWIEPGTVVHDPASCGGAPEPTSQFEHSSIPATVKRIFGLKEFLTRRDAWAGTFDTVLTRAAPREDCPATLPEPPRLRAAEAEEHREVSEFQAELVQLGAALNGDHDGEGYDPEVFVRGMTVAGAAQYCRDAFDRFREECHRCRDGGMDGSHVPMLQPASASASSSAPAADPPATAPAPATPSALSKLCGCFPCFNAS

>OsNPC4

MAAAAAGGKIKTVVVLVMENRSFDHMLGWMKSLNPEIDGVTGDEINHLDAADPTSRAIRFGDGAEYVDPDPGHSMQAIYEQVYGTPFVDARATPITPPGVPSPPMAGFAQQAEKEKPGMADTVMNGFRPEAVPVYRELVRQFAVCDRWFASNPASTQPNRLFVHSATSHGLVSNDTKLLVAGLPQRTIFDSLHDAGFSFGIYYQYPPSTLFYRSLRQLKYAGNFHPFDLAFRRHCAEGKLPNYVVVEQRYFDLKMLPGNDDHPSHDVSEGQRFVKEVYEALRGGPQWEEALLVVTYDEHGGFYDHVPTPVDVPSPDGIVSAAPFFFEFNRLGVRVPALFISPWIEPGTVVHRPSGPYPTSEFEHSSIPATVKKLFNLKSFLTNRDAWAGTFDVVLTRDAPRTDCPATLPEPVKMRPATEA

AEQAALTEFQEELVQLGAVLNGDHADEDVYPRKLVEGMTVAEAASYCNAAFKAWMDECDRCRKCGEDGSHIPTVVKPPPPPSTSSSGSSSFASKLLSCFACGRPNKN

>OsNPC5

MWPLSPHRRHLQQHGHSGAMGRRLLLLFLMLAQAPNSNGDSKIKNVVVLALENRSFDHMLGWMQRLLGLPIDGLTGAECNPAPGPGPADSLLHCVSPDADLVVPDDPAHAFEDVLEQLLGFRPNDSTGAAASPSDMSGFVRSAVSVSALLTDAVMRGFTPSRLPAFSALASSFAVFDRWFSSIPGPTQPNRLFLYSATSHGAVAHDKWNLLRGYPQRTIFDSLAADALDYRVYFKTIPTTLFYRRLRTVANAARGTFRRYDAAFRDHARRGLLPALSVIEPRYFDLTGTPADDDHPAHDVANGQRLVKDVYEALRAGPQWNHTLLIITYDEHGGFYDHVPPPNVGVPSPDAIRGPLPFFFRFDRLGVRVPTIMVSPWIRKGTVVGRPPGGPTPTSEYEHSSIPATIKKIFNLSSDFLTRRDAWAGTFEHLFTDLDEPRTDCPETLPEIPPPSSSSSSTKKEDGGWLSDFQRELVQLAAFLNGDYMLSSFAQEYESRMTMTVKQADAYVRRAVKSFLEASKRAKRLGANDSAIVTMRPSLTTATTCCP

>ZmPI-PLC1

MGTTYKCCFIFKRRFHTRDAPPPEDVRALFSVHAGGGPHMGADDLRRYLVATGQEEAEVERLLEQIVQGRARVPRLARPLLSLDDFHRYLFSHDLNPPIRHPQVHHDMTRPLSHYFIYTGHNSYLTGNQLSSDCSDTPIIMALERGVRVIELDMWPNSTIDDINIVHGRTLTTPVSLIKCLRSIKKHAFVASPYPVIITLEDHLPSQLQEKVAKMVVEVFGNILYYPPDDTHELKELPSPEQLKGRVLLSTKPPKEYLETKLSDSTKEGDADLHLGKGTGDDAVWGKEVPDFRTEIQSAEKDDENDDVDDEEEQKLQPHIAPQYKHLITIRAGKPKDSLADALKSDPEKVRRLSLSEQQLAKAAKDHATEIVRFTQRNILRIYPKGTRVTSSNYNPFVGWVHGAQMVAFNMQGYGRALWLMHGFYKANGGCGYVKKPDFLMQTEPQVFDPREPQPVKKTLKVKVYMGDGWRMDFKQTHFDQYSPPDFYTRVGIAGVPADSVMKKTNAIEDNWTPVWEKEFSFPLTVPELALLRVEVHEYDMSEKDDFGGQTVLPVSELRPGIRAVALFDRKGMKYNHVKLLMRFEFA

>ZmPI-PLC2

MGTYKCCIFFTRRFALGDTSTPEDVRALFSLYAGGSPYMLADDLRRYLAAWGGADGEVPEQIIDRILQDRSRTPRFGRPALTVDDFLHFLFSEDLNPPLRYSKVHQDMNAPLSHYFIYTGHNSYLTGNQLSSDCSDVPIIKALQLGVRVIELDIWPNSCKDDIDVLHGRTLTAPVSLIKCLRSIKEYAFVASPYPVIITLEDHLTPDLQERVAKMVLEVFGDILYYPESKHLQEFPSPEALKGRVMLSTKPPKEYLEAKGDTMKEKEIEARIKRGEREAAAWGVEVPDIQDEMQVADKDEDELLYRERGADGDDGKETKHVAAEYKHLITIKAGKPKGPLVDAIKNDPDKVRRLSLSEQELAKVAAHHGPNIVSFTHRNMLRIYPKGTRFNSSNYNPFLGWVHGAQMVAFNMQGYGRALWLMHGFYKANGGCGYVKKPDFLMQTCPDGKVFDSKADLPVKATLKVKVYMGEGWQKDFKQTHFDSYSPPDFYVKVGIAGVPLDSVMRKTKAVEDSWVPVWEEEFAFPLTVPEIAVLRVEVHEQDVNEDDFGGQTALPVAELRPGIRAVPLFDHKGLKFKSVKVLMSFEFA

>ZmPI-PLC3a

MGSYAYKYCMCFTRKFRSPDAQPPPDVRAAHLSFASDAHALRRFVAGVQGESPADVDRILAMLSGGHSHGIARLVTRSPAASTPTLEDFLAFLFSPDLNPPIAHQVHQDMSAPFSHYFVFTGHNSYLTGNQLNSDSSDVPIVKALQGGVRVIELDMWPNPSKDNVDILHGGTLTAPVEMIKCLKSIKEYAFCASNYPLVITLEDHLTPDLQAKVATMLTETFGDLLFVPNPDPMKEFPSPASLMKRIIISTKPPQEYKEFLKAENNRSGSGNIAELPDQGSLRRIDSNADESDGKDELDEQDEEDSDSDDPKFQQDTACEYRKLITIQAGKPKGHLRDALKVDPDKVRRLSLSETQLAKATISHGAEVIRFTQKNILRVYPKGTRVNSSNYDPMNAWTHGAQMVAFNMQGHDKALRLMQGFFRANGGCGYVKKPDFLLRTGPNGEVFDPKASLPVKKTLKVKVYMGDGWRMDFSKTHFDAFSPPDFYTRVGIAGVKADSVMKKTRVIEDQWVPMWDEEFTFLLTVPELALLRVEVQEYDMSEKHDFGGQTVLPVWELKQGIRAVPLHDRKGVRYKSVRLLMRFDFV

>ZmPI-PLC3b

MGSYAYKYCMCFTRKFRSPDAQPPPDVRAAHLSFASDAHALRRFVAGVQGESPADVDRILAMLSGGHSHGIARLVTRSPAASTPTLEDFFAFLFSPDLNPPIAHQVHQDMSAPFSHYFVFTGHNSYLTGNQLNSDSSDVPIVKALQRGVRVIELDMWPNPSKDNVDILHGGTLTAPVEMIKCLKSIKEYAFCASNYPLVITLEDHLTSDLQAKVATMLTETFGDLLFVPNPDPMKEFPSPASLMKRIIISTKPPQEYKEFLKAENNRSGSGNIAELPDQGSLRRIDSNADESDGKDELDEQDEEDSDEDDPKFQQDTACEYRKLITIQAGKPKGHLRDALKVDPDKVRRLSLSETQLAKATISHGAEVIRFTQKNILRVYPKGTRVNSSNYDPMNAWTHGAQMVAFNMQGHDKALRLMQGFFRANGGCGYVKKPDFLLRTGPNGEVFDPKASLPVKKTLKVKVYMGDGWRMDFSKTHFDAFSPPDFYTRVGIAGVKADSVMKKTRVIEDQWVPMWDEEFTFLLTVPELALLRVEVQEYDMSEKHDFGGQTVLPVWELKQGIRAVPLHDRKGVRYKSVRLLMRFDFV

>ZmPI-PLC4

MTTYRVCCFLRRFRAASNEPSEAVGDVFRAYADGGGGLLGEEALRRFLREVQRETDAGADAAAKEVVAFAAEHRLLSKGRGLTAEGFLRWLFSDANAALDPRRGVYQDMGLPLSHYFIYTGHNSYLTGNQLSSGCSERPIVKALHDGVRVIELDLWPNAAKDDVEVLHGRTWTSPVELIRCLEAIKEHAFATSPYPVILTLEDHLNRDLQAKVAKMLKETFGEMLYVSESEHMAEFPSPDELKGKIVISTKAPKECFQTKSSKDAAATEEEEGVWGEEISDDKATARQMSEQSSGKYTADEETAGAGAEDEMEVEAEAEAEKKSRQGTEDNEYRRLIAIQLTRRKHDIEQDLRVDPDKVTRVSLGEKAFEKAIVSHGDHVVRFTQRNLLRIFPRSTRITSSNYNPLMGWRYGVQMVAANMQGHGRKLWLTQGMFRANGGCGYVKKPDALMRSKPLDFDPRAELPVKTRLRVTVYMGDGWRFDFRKTHFDRCSPPDFYARVGVAGVPADTRMEQTRVAMDSWVPAWDHEFGPFPLAMPELALLRVEVHESDNHQKDEFGGQTCLPVWELRPGIRSVRLSDHKGQPLPSVKLLMRFDFFPASDSNSTQ

>ZmNPC1a

MAAVARRGPGARLLAALLLLALVVSGHCLDAHHRGLKRRRRKHEIHSPVKTVVVVVMENRSFDHILGWLRRTRPDIDGLTGRESNRLNASDPASPEIFVTDEAGYVDSDPGHGFEDIREQIFGSADTSAVPPPMSGFAQNARGMGLGMPQNVMSGFKPDAVPIYASLADDFAVFDRWFASVPTSTQPNRLFVHSATSHGLTFNARKDLIHGFPQKTIFDSLEENGLSFGIYYQNIPATLFYQSLRRLKHLIKFHQYSLKFKLHAKLGKLPNYVVIEQRYFDCEMFPANDDHPSHDVARGQRFVKEVYETLRASPQWNETALIITYDEHGGFYDHVPTPVVGVPQPDGIVGPDPYYFKFERLGVRVPTFLISPWIEKGTVIHEPNGPQDTSQYEHSSIPATVKKLFNLHSNFLTKRDAWAGTFESYFKIRKTPRTDCPEKLPEVTKSLRPFGPKEDSSLSEFQVELIQLASQLNGDHVLNTYPDIGRTMTVGEANRYAEDAVARFLEAGRIALRAGANESALVTMRPALTSRASMSSGLSSEL

>ZmNPC1b

MFPANDDHPSHDVARGQRFVKEVYETLRASPQWNETALIITYDEHGGFYDHVPTPVVGVPQPDGIVGPDPYYFKFERLGVRVPTFLISPWIDKGTVIHKPNGPQDTSQYEHSSIPATVKKLFNLHSNFLTKRDAWAGTFENYFKIRRTPRTDCPEKLPEVTKSLRPFGPKEDSSLSEFQVELIQLASQLNGDHVLNTYPDIGRTMTVGEANRYAEDAVARFLEAGRIALRAGANESALVTMRPALTSRASMSSGLSSEL

>ZmNPC2

MSAARRRSPIPVLLFFLLLHSASPSPIKTVVVLVMENRSFDHMLGWMKRLNPEIDGVTGREWNPANASDPAAGRVYFRDGAAYVDPDPGHSFQEIRQQVFGSDDDAADNPPRMDGFAQQARSIGGGAMSDAVMRGFNPADVAVYRELVSQFAVCDRWFASVPSSTQPNRLFVHSGTSGGATSNNPTLLAEGYPQRTIFDNLHDAGLSFGVYFQDVPAVLFYRNLRKLKYLLDFHPLRPSFADHARRGTLPNYAVIEQHYLDSKLDPANDDHPSHDVYQGQMLVKYVYETLRASPQWNQTLLVITYDEHGGFFDHVPTPVAGVPSPDGIVGPPPYNFTFDRLGVRVPAILVSPWIDRGTVVHGPHGPTPTSQYEHSSIPATVKKIFGLPQDFLTRRDAWAGTFEGVVQGRTEPRTDCPEQLPTPTRIRQTEADEEAKLSEFQQEIIQLASVLNGDYHLATLQDRIKNDMNVREGIDYMKAAVKRYFQAGAFARRMGVDGDQIVKMRPSLTTRIQRP

>ZmNPC3

MAAAEPVPVPVPAAAAGTNNKIKTVVVLVQENRSFDHMLGWMKSLNPDIDGVTGAETNHVDASDPASHAVRFSDGAEYVDPDPGHSMQAIYEQVYGTPFVDAATTPATPPGVAPSMSGFAQQAEKERPGMSATVMSGFRPDAVPVYRDLVREFAVCDRWFASNPASTQPNRLFVHSATSHGLVSNDSRALVAGLPQRTIFDALHDEGLSFGVYYQYPPSTLFYRSLRQLKYAASFHAFDLHFARHCREGRLPSYVVVEQRYFDLRFLPGNDDHPSHDVAEGQRFVKEVYEALRSGPQWPQTLLVVTYDEHGGFYDHVPTPAGAGVVPSPDGIVSAKPFSFAFDRLGVRVPALLVSPWIEPGTVLHGPPSGPCPTSEFEHSSIPATVKKLFNLRSFLTRRDAWAGTFDCVLTRDIPRTDCPLTLPEPVKLRRTAAAEHAPLSEFQEELVQLAAVLNGDHTKESYPQGLVEGMTVAEAARYCVDAFKAFLDECEKCKDRGGEDGSHIPTVKPTTTGKEKDKSKSSFASKSLSCLPCCVRPSSS

>ZmNPC4

MAAAEPVPVPVPAAAAGTNNKIKTVVVLVQENRSFDHMLGWMKSLNPDIDGVTGAETNHVDASDPASHAVRFSDGAEYVDPDPGHSMQAIYEQVYGTPFVDAATTPATPPGVAPSMSGFAQQAEKERPGMSATVMSGFRPDAVPVYRDLVREFAVCDRWFASNPASTQPNRLFVHSATSHGLVSNDSRALVAGLPQRTIFDALHDEGLSFGVYYQYPPSTLFYRSLRQLKYAASFHAFDLHFARHCREGRLPSYVVVEQRYFDLRFLPGNDDHPSHDVAEGQRFVKEVYEALRSGPQWPQTLLVVTYDEHGGFYDHVPTPAGAGVVPSPDGIVSAKPFSFAFDRLGVRVPALLVIGTDRHGTVDAVHAVTLPEPVKLRRTAAAEHAPLSEFQEELVQLAAVLNGDHTKESYPQGLVEGMTVAEAARYCVDAFKAFLDECEKCKDRGGEDGSHIPTVKPTTTGKEKDKSKSSFASKSLSCLPCCVRPSSS

>ZmNPC5

MATGSPRRRPLPLLLLFLLLFAANGRANAARPGGPSPIKNVVVLALENRSFDHMLGWMRRLLGLPVDGLNGTECNPNSNSTSSICVSADADLVVPDDPGHSFEDVLEQVFGNVSAAAAQPQPSMSGFVRSALSVNAVLSSAVMRAFRPSLLPAFSALAPQFAVFDRWFSSIPGPTQPNRLFLYSATSRGAVAHDKLDLLLGYPQRTIFESLAADGHDFAVYFKTIPTVLFYRRLRALRYAARAFHRYDAAFKEHARRGVLPALSVIEPRYFDLTGTPADDDHPAHDVANGQRLVKDVYEALRASPQWNQTLLIVTYDEHGGFYDHVATPTAGVPSPDGIRGPPPFFFKFDRLGVRVPTIMVSPWIKKGTVVGRALGPTDTSEFEHSSIPATIKKIFNLSSDFLTKRDAWAGTFDHIFTELDQPRTDCPETLPEVPFERPTPPKEHGWLSDFQRELVELASFLNGDYMLTSLAQENRKKKM

TVKQADAYVRRAITSFLQASKQAVRLAANESAIVTMRSSLTSKSTRSSSP

>BdPI-PLC1

MGTYKCCFCFRRRYRWTDAPPPDDVRSLFAHHSGGAATMGADGLRRYLESKGTGAEDDNSEAAADRLLDQIRHGQRGARIPRVGRPLIALEEFHRFLFSDDLNPPIRRPQVHHDMTAPLSHYFIYTGHNSYLTGNQLSSDCSDIPIIKALQKGVRVIELDMWPNSTRDDINILHGRTLTTPVSLLKCLRSIKEYAFVVSSYPVIITLEDHLPPDLQDKVAKMVLEVFASILYYPDTEHIKELPSPEDLKGRVLLSTKPPKEYLEAKAGGTIKDGDADPNLGKGAGEDAAWGKEVPDFKTEIQSAKQEDDVSEHHRDDDDEDDDDDEEEQKMQQHLAPQYKHLITIKAGKPKGTTADALRCDPDRVRRLSLSEQELAKAVVHHGTEIVRFTQRNLLRIYPKGTRVTSSNYNPFLGWVHGAQMVAFNMQGYGRALWLMHGFYKANGGCGYVKKPDFLMQSDVFDPKKRQPVKKTLKVKIYMGDGWRMDFKHTHFDQYSPPDFYARVGIAGVPADSVMKKTRTIEDNWVPVWEEEFAFDLTVPEIALLRVEVHEYDMSEKDDFGGQTVLPVSELQPGIRAVALFDRKGNKFNNVKLLMRFEFV

>BdPI-PLC2

MSKRAERKAGEPRQERGEAEAAAMGTYKCCIFFTRRFALSDASTPEDVRTLFSRFSSGAPYMGVDELRRYLAATGGADGDSGGDGDLEAAERIIDRALQGRTRTPRFGKPSLTVDDFHNFLFSEDLNPPIRHPKVHHDMSAPLSHYFIYTGHNSYLTGNQLSSDCSDIPIIKALQIGVRVIELDMWPNSSKDDIDILHGRTLTAPVSLIKCLTSIKEYAFVASPYPVIITLEDHLTSDLQAKVAKMVLEVFGDILYYPESKHLQEFPSPEALKGRVILSTKPPKEYLEAKGGTMKERDIQPQFKKGENEQSAWGIEVPDIQDEMQNADKNQEDLLYRERDVDEDDEQKMRQHAPLEYKHLITIKAGKPKGSLVDALKSDPEKVRRLSLSEQELAKVAARHGPNIVSFTQRNLLRIYPKGTRFNSSNYNPFLGWVHGAQMVAFNMQGYGRALWLMHGFYKANGGCGYVKKPDFLMQTCPDGKVFDPNADLPVKTTLKVKVYMGDGWQNDFKQTHFDSYSPPDFYAKVGIAGVPLDSVMRKTRAVEDSWVPVWEEEFTFPLTVPEIALLRVEVHEYDVNEDDFGGQSVLPVSELRPGIRAMPLFDHMGMKFKSVRLLVSFEFV

>BdPI-PLC3

MGSYAYRYCMCFTRKFRSPDAHPPPDVRAAHAAAASADEGAGLRRFLADVQREDPSEADRILAALAGGGGGGIARLVGRSPALPSLDDFFGFLFSPDLNPPIPDKVHQDMSAPISHYYVFTGHNSYLTGNQLNSDSSDIPIIRALQRGVRVIELDMWPNSAKNCVEILHGGTLTAPVEIIKCLKSIKEHAFCASTYPLVITLEDHLTADLQAKVAEMITETFGDLLYVPSSDALNEFPSPEALMKRIIISTKPPQEFREFLKAQGNQNNNGNTANLSDEGALRRLDSNADDSDGKDDLDEEDDEESEEEDPKFQPDTACEYRKLITIHAGKPKGHLRDALKVDPDKVRRLSLSETQLTKATASHGADIIRFTQKNILRVYPKGTRINSSNYDPINAWTHGAQMVAFNMQGHDKSLRLMQGFFRANGGCGYVKKPDFLLKTGPNGEVFDPKANLPVKKTLKVKVYMGDGWRMDFSKTHFDAFSPPDFYARVGIAGVKADCVMKKTKTIEDQWVPVWDEEFTFPLTVPELALLRVEVQEYDMSEKHDFGGQTCLPVMELKQGIRAVPLHDRKGNRYKSVRLLMRFDLI

>BdPI-PLC4

MTTYRVCCFLRRFRPASSEPSAAIAAVFRSYASAASAGDVLGEEALKRFLREVQGEEGGSAGVGDEDEDGGVEALVREIMAFAAQQKLLKKGGGGGGIAAEGFHRWLCSDANAAIHPRRGVHQDMGQPLSHYYIYTGHNSYLTGNQLSSGSSVAPIVKSLLDGVRVIELDLWPNAGKDDVEVLHGRTWTSPVGLGECLDAVKEHAFVSSPYPVILTLEDHLTPHLQAKVAKMIKETFGDMLHISQSETMAEFPSPDDLKGKIIISTKQPKEYLQTKSSKEDSQNGDKEEEESVWGEEIPDNKAQPAVARQMSEPDNGQGVEEEDEMDKKVQPGVDGEYKRLIAIQLTRRKHDMNEDLKVDPEKVTRMSLGEKAYEKATITHGADIIRFTQRNLLRIFPRTTRITSSNYNPLMGWRYGAQMVAANMQGHGRKLWLTQGMFRANGGCGYVKKPDFLMNSDPDKMFDPRSKLPVKTRLKVTLYMGDGWRFDFHKTHFDKFSPPDFYARVGIAGVAADTRMEESKVVMDNWIPTWDHEFVFSLAVPELALLRIEVHEADNHQKDDFAGQTCLPVWELRQGIRSVRLCARDGELLRSVKLLMRFEFA

>BdNPC1

MAGQRRGGRHLLVGALLLALVVSAHCLDSHHGRGLKRRRRKHEIHSPIKTVVVVVMENRSFDHVLGWLRGQRPDIDGLTGKESNRLNASDPSSPEIFVTDKAGYVDSDPGHGFEDIREQIFGSADTSAVPPPMSGFAQNARGMGLGMPQNVMSGFTPDSVPVYASLAEEFAVFDRWFASVPTSTQPNRLFVHSATSHGLTFNARKDLINGFPQKTIFDSLEENGLSFGIYYQNIPATLFYQSLRRLKHLVKFHQYSLKFKLDAWRGKLPNYAVIEQRYFDCKEFPANDDHPSHDVARGQRFVKEVYETLRASPQWNETALIITYDEHGGFYDHVPTPVVGVPQPDGIVGPDPYYFKFERLGVRVPSFLISPWIEKGTVIHEPNGPTPTSQYEHSSIPATVKKLFGLHSNFLTKRDAWAGTFENYFKIRKTPRTDCPEKLPEVLKSLRPFGPDEDKSLSEFQVELIQLASQLNGDHVLNTYPDIGKTMTVVEANRYAEDAVARFLEAGRIALRAGANESALVTMRPALTSRAAMSTGLSAEL

>BdNPC2

MAAPPRGPRRRPRPHTLPAAAILVLSLLLLSPPSSSSSAAAGPTSPIKTVVVVVMENRSFDHMLGWMKSLNPAIDGVTGAEWNPANASDPSSPRVYFRDGAQYVDPDPGHSYQEIRQQVFGSDDASGPAKMNGFVQQATSIGGGNMTDAVMHGYKPDAVAVYKELVSQFAVCDRWFASVPSSTQPNRLFVHSGTSGGATSNNPELLAEGYPQRTIFDNIYDAGLSFGVYFQDVPAVLFYRNLRKIKYLLKFHPFHNTFRDHAQSGNLPNYAVVEQHYMDSKSHPANDDHPSHDVYQGQMFIKEIYETLRASPQWNETLMVITYDEHGGFFDHVPTPVDNVPSPDGIVGPPPYNFEFNRLGVRVPTIFISPWIEKGTVVHGPNGSPTPNSQYEHSSIPATVKKLFNLPQDFLTRRDAWAGT

FESVVKTRTEPRTDCPETLPMPTRIRQTDANEEAKLSSFQQEIVQLAAVLNGDHQLTSLQERIRDRMNVREGTAYMRRAVRRFFEAGLSAKRMGVANDEQIVKMRPSLTTRTSSDIDQEHP

>BdNPC4

METFLELLLLRTPFQQENRSFDHMLGWMKSLNPEIDGVTGTETNHVIAGDASSKAVVFSDKSEYVDPDPGHSIQAIYEQVYGTPFVDATATPITPSSPATMAGFAQQAEKEKPGMSETVMSGFKPDAVPVYKELAKEFAVCDRWFASNPASTQPNRLFVHSGTSHGLVSNDTKTLVAGLPQKTIFDSLDDAGLSFGIYYQFPPATLLYRNLRQLKYLDNFHQYDLSFRRHCREGKLPNYVVVEQRYFDLANIGLPGNDDHPSHDVGEGQRFVKEVYEALRAGKQWEETLLVVTYDEHGGFYDHVPPPSGAGVVPSPDGIVSAPPFFFNFDRLGVRVPAFFISPWIEPGTVVHRPAHGPEPTSEFEHSSIPATVKAIFGLPSFLTKRDAWAGTFDTVLTRQTPRKDCPTKLPEPVKMKRLTAVAAAAPVISEFQAELVQLAAALNGDHAREDYPEKLVEGMTVPEAVSYCNDAFKAFRDECERCKSCGMDGSHVPILPLPAPPAPPAPEKKNSFPSKILACFACGRS

>BdNPC5

MGLPPPLPSLLLLIILLVAGSITHNSGNAAASSSSSPIKNVVVLALENRSFDHMLGWMRRLLGLPIDGLTGAECNPFTTSSSPSLPPICVSSDADLVVPSDPGHSFEDVLDQVFGFRPPAPNPRNQSQSPPPNPTMSGFVRSALSVDGARLPSAVMRGFTPRLLPSFSALAAGFAVFDRWFSSIPGPTQPNRLFLYSATSHGAVAHDKLRLLAGYPQRTIFDSLADESLPFAVYFKSIPTTLFFRRLRTVRAAAGSFHFYDDTFRSHARTGTLPALSVIEPRYFDVPSAGAPADDDHPAHDVAQGQRLVKDVYEALRAGPQWNSTLLIVTYDEHGGFYDHVATPVAGVPSPDAVRGPLPFFFKFDRLGVRVPTIMVSPWIKKGTVVGRPPNGPTATSEYEHSSIPATIKKIFNLRSDFLTKRDEWAGTFEHIFTELKEPRTDCPETLPEVPFERTRPAKEHGLLSDFQRELVELAGFLNGDYMLASFAQEAQKNMTVKQADAYVRRAITSFLQASKQARRLGANESAIVTMRSSLTSKSTTSSP

>GmPI-PLC1

MGNNIATYNKYKSLLFFIRKYKFTELLLRATELLPPQDLKEAFSKFTGGGSYMSAEQLHGFLMEHQGQVRKTCQETVNKVDQNREHEITLDELFRFLLHDDSNAPLKAEVHHDMGAPLSHYFIYTGHNSYLTGNQLSSDCSEEPIIKALKRGVRVIELDLWPTYNKHDIKVDHGWTLANPASVIICLESIKEYGFVASQYPVIITIEDHLTTDLRAKFAEMATQIFGEMLFYPGADCSCLTEFPSPESLKNRVIISTKPPKERFKSYRIKDNPMLNESESSEEESWENESPDSNKNEVETEDMNGSDHDEGNESACECARKPYQVCSPDYKHIITIHNTKLKGCLKDKLKTDGEVRRLSWSEKTLEKASESHGTDILRFTQKNILRVYPSAMRVKSSNFKPNIGWMYGAQMVACNMQGLGKSLWLMQGMFRANGGCGYVKKPQFLMQKYHCDNEFDPTRIQSVKKKTFKVKVYMGHGWSSDLSPTHFDKCSPPDFYTKVCIVGMPDDVAKKKTKVMMDNWFPVWDEEFEFSLIVPELALLLIQVKDKDPGKDDFAGQTCLPVSELKHGFHSVPLYNKKGEKFKSVKPLMQFQFE

>GmPI-PLC2

MGTVTYSYKMFKYFNRKFDVSEQAPPPDVKEAFSAFSHGAASSMSAHQLLRFLHDHQRETDCTAEDSNRILDSIIQSRKQTNDTNSERHHHPDNNKNGLTLDEFFRFLFLLDFNDPLKSQVHHDMNAPLSHYFIYTGHNSYLTGNQLSSVCSDVPIIKALQRGVRVIELDLWPNSTKDDIDVVHGRTLTAPVSLIQCLKSIKEYAFVKSNYPVIITLEDHLTPFLQARVAEMIAQVFGDMLYFPHEDPLTEFPSPESLKGRILISTKPPKEYLESKQFKDSDSERESNEEGSLSPCIIPELEVVDEKSNGNDLDEEGLSARDKKSDQQSAPEYKRLITINAGKPKGHVKDHLNNVGGVKRLSLSEHELEKASATYGSDIVRFTQKNIIRVYPKGTRVTSSNYRPHIGWMYGAQMVAFNMQGHGKSLWYMQGMFRANGGCGYVKKPAFLTEKGPHNEVVDPKRALPEKKSLKVKVYMGNGWSSDFSKTHFDTFSPPDFYTKVCIVGVPADKANKKTKVIQDNWFPVWDEEFEFPLTVPELALLRIEVREYDKHEKDDFGGQTCLPVSELRSGFRAVPLFDQKGEQLKSVKLLMRFQFVDSVPTSQ

>GmPI-PLC3

MTSKQTYSVCFCWRRRFKLALAEAPSEIKTLFNEYSENELMTPSHLKRFLVDVQRQEKATEEDAQAIIDSFRHFHRRGAGLNLETFFKYLFSDDNPPLLPSHGVHHDMTLPLSHYFIYTGHNSYLTGNQLSSDCSDVPIINALKKGVRVIELDIWPNASKDSIDVLHGRTLTTPVELIRCLRSIKDHAFVASEYPVVITLEDHLTPDLQAKVAEMVTQTFGDILFTPNSESVKEFPSPESLKKRIIISTKPPKEYLEAKEKEKGDDSQHEKEKGDDSEHGKASGEDEAWGKEVPSLKGGTIEDYKDNNVDEDLNDEEEFDESDKSHHNEAPEYRHLIAIHAGKPKGGLVECLKVDPEKVRRLSLSEQQLEKAAINYGQQIVRFTQRNILRVYPKGTRIDSSNYNPLIGWMHGAQMVAFNMQGYGRSLWLMHGMFRANGGCGYVKKPNFLLETGPDDEVFNPKAKLPVKTTLKVTVYMGEGWYYDFKHTHFDQYSPPDFYTRVGIAGVPNDTIMKRTKAIEDNWLPTWNEAFEFPLTVPELALLRIEVHEYDMSEKDDFGGQTCLPIWELRSGIRAIPLHSQKGDKYNTVKLLMRFEFINN

>GmPI-PLC4

METYIIKVLNFLTKKGEVYKAEPPLDLKEAFSKFAGGENQMKKDQLLRFMVEHQGENNISTMEDLDKIVEKFLQLRNSCSSTKTSSTRIVDVYRKQGLSLNDFIDFLILDDFNGPLKDKVHHDMNAPLSHYFMYTGHNSYLTGNQLTSESSDEPIIEALKQGVRVIELDLWPSKSSNNDGIKVVHGRTFTTPVPVSKCLQSIKDYAFHKSDYPMATEIFGEMLYCPETDYLTEFPSPESLKKRIIISTKPPKEYQQSDSIRKPMPNGSEPSEEESWGPELPDSVAKLKTEDRNSSDEDQEDINTSDNKSNQQGVRQYKHLIAIHGGKSKGSMKNRLKDDVKVRRLSLSEKKLKSASESHGADLIRFTQRNILRVFPKGERVKSTNFRPYIGWLYGVQMVAFNMQGHGKSLRLMQGMFRANGGCGYVKKPEFLIRTLPHEEVFDPKKPPSRKQILKVKVYKGDGWRLDFSPTHFDRFSPPDFYTKVSIVGVPADCAKNKTRVKTDTWYPVWDEEFEFKLRVPELALLRIEVKDKDKGKDDFAGQTCLPLSELRHGFRSVPLYDRKGKKYKSVTLLMRFKLETLN

>GmPI-PLC5

MALFLCPQVCSIHIVTARSLQLQSPFHSAAIAISFLKHTHRHQSLSLIHSVYTIMASHNYKVFSCFNRKFTVTEPGPPPDVERAFSEFAAGASSLSGDHILRFLAEHQGDVDCTAADSERMLQQSRKEDRESGMDLHDFFRFLLHDDFNSPIKSQVHHDMNAPLSHYFIYTGHNSYLTGNQLSSDCSDVPIIKALQGGVRVIELDLWPNSDKDDIEVVHGRTLTTPVSLIQCLKSIKEYAFVKSHYPLIITLEDHLTPDLQAKAAEMATQVFGELLYYPQTDSLMEFPSPESLKGRILISTKPPKEFLESKECDDKDNEKESADEVLSLPDQTSERETDDKSAPEYKRLITIHAGKPKGDIQDELKAAGAVRRLSLSEQALEKASESYGADIVRFTQNNILRVYPKGTRLNSSNYKPHIGWTYGAQMVAFNMQGYGKSLRYMQGMFRANGGCGYVKKPEFLIEKGPYNEVFDPKRTLPVKKTLKVKVYMGTGWSLDFSQTDFDTYSPPDFYVKVCIVGVPADMAKKKTCVISNNWFPVWDEQFDFPLTVPELALLRIEVRENDKSQKDDFGGQTCLPVSELKSGFRSVPLYDEKGDKLKSVKLLMRFQFR

>GmPI-PLC6

MSSKQTYSFCFCFRRRFRIPVSEAPPEIRTLFDGYSDENGVMTATHLRSFLAEVQREDNATEEEAQAIIDGHKHLSIFHRSGLNLESFFNYLFSHHNNPPLLPSLGVHQDMSSPLSHYFIYTGHNSYLTGNQLSSDCSDVPIINALEKGVRVIELDIWPNESKDNVDVLHGRTLTSPVALIKCLRSIKQHAFVASEYPVVITLEDHLTPDLQAKVAEMITQTFGDILFAPTSESLKEFPSPESLKGRIIISTKPPKEYLEAKEVQEKEEESQQEKPADDEEAWGKEVPSLRGGTISDYKNIEDDDVLDDEEDIDEAEKSRQDAADEYRRLIAIHAGKPKGGLTECLKVDPDKVRRLSLSELQLEKAAETHGKEIVRFTQRNILRVYPKGTRITSTNYNPLIGWMHGAQMVAFNMQGYGRSLWLMQGMFKANGGCGYVKKPDLLLKVGPNNEVFDPRSHLPVKTTLKVTIYMGEGWFLDFKHTHFDKFSPPDFYARVGIAGVPNDTVMKKTEKVEDNWSPSWNQVFKFPLAVPELALLRVEVHEYDMSEKDDFGGQTCLPVWELRSGIRAVPLYSRKGDKYANVKLLMHFEFI

>GmPI-PLC7

MTSKQTYSVCFCWRRRFKLALAEAPSEIKTLFEEYSENEFMTPSHLKRFLVEVQRQEKATEEDAQAIIDSFRHFPRRGAGLNLETFFKYLFSDDNPPLLPSHGVHHDMTLPLSHYFIYTGHNSYLTGNQLSSDCSDVPIINALKRGVRVIELDIWPNASKDNIDVLHGRTLTTPVELIRCLRSIKDHAFVASEYPVVITLEDHLTPDLQAKVAEMVTETFGDILFTPNSESVKEFPSPESLKKRIIISTKPPKEYLEAKEKEKGDDSQHEKEKGDDSQHGKALGEDEAWGKEVPSLKGGTIEDYKDYNVDEDLNDEEEFDESDKSHHNEAPEYRRLIAIHAGKPKGGLAECLKVDPDKVRRLSLSEQQLEKAAINHGQQIVRFTQRNILRVYPKGTRIDSSNYNPLIGWMHGAQMVAFNMQGYGRSLWLMHGMFRANGGCGYVKKPNFLLETGPDDEVFNPKAKLPVKTTLKVTVYMGEGWYYDFKHTHFDQYSPPDFYTRVGIAGVPNDTIMKRTKAIEDNWLPTWNEVFEFPLTVPELALLRIEVHEYDMSEKDDFGGQACLPIWELRSGIRAIPLHSQKGDKYNTVKLLMRFEFINN

>GmPI-PLC8

MGTVTYSYKMFKYFNRKFAVSEQAPPPDVKEAFSAFSDAAAASMSADQLLRFLHDHQRETDCSAEDSNRILDSIIQSRKQNDTNAECDHHTDNNNNGLSLDEFFRFLFLVDFNDPLKSQVHHDMNAPLSHYFIYTGHNSYLTGNQLSSDCSDVPIIKALQRGVRVIELDLWPNSTKDDIDVVHGRTLTAPVSLIQCLKSIKEYAFVKSDYPVIITLEDHLTPFLQAKVAEMIAQVFGDMLYFPQADSLTEFPTPESLKGRILISTKPPKEYLESKQFKDSDSERESTEEGSLSPCVIPELEAVDEKLNGSDLDEEGLNARDKKSDQQSAPEYKRLITIHAGKPKGHVKHHLNNVGGVKRLSLSEQELEKASATYGSDIVRFTQKNIIRVYPKGTRVTSSNYRPHIGWMYGAQMVAFNMQGHGKSLWYMQGMFRANGGCGYVKKPAFLIEKGPHNEVFDPKRALPVKKTLKVKVYMGNGWSSDFSKTHFDSFSPPDFYTKVCIVGVPADKANKKTKVIQDNWFPVWDEEFEFPLTVPELALLRIEVREYDKHEKDDFGGQTCLPISELRSGFRAVPLFDQKGEQLKSVKLLMRFQFK

>GmPI-PLC9

MATTSRHPSRNKTPKQHFKVCFCFRRMFRLKGTEPPDEINTVFEEYSLDGIMSMHDLGNFLVHFQGEEEGDSINKHAQTIFDSLKHLNIFHRKGIHVEAFFRYLLSDLNGPLAEVHHDMKFPLAHYFLYTGHNSYLTGNQVSSASSTSAIIKALKKGVRVIELDLWPNSRGDDVLVHHGGTLTSSVKLKACLKAINDYAFFASPYPVVITFEDHITQFLQAKVAQMVDDIFGDILFRPEYSQQMTQFPSPEQLKGKILISTKPPESPESQDQRVREEAHRLEYDDDRTRVNYKDDLEEQEEEDDTLEYRDLISIRAGKPKGKLRNWLIDHEQVRRLSLSEQELEDIAKNHGTDIVRFTQRNLLRIYPKGTRLDSSNYDPMIGWMHGAQMVAFNMQGGGHHLRYMEGMFRANGGCGYVKKP

DILLNVGPNNEVFDPRTIRPVQKILQVLVYMGEGWRSDFGPTHFDFYSPPDFRVKVGIHGVPADKDTKYTRTVEDDWVPVWNEEFSFPLTVPELALLYIKVVERDFSGRHDFGGQTCLPVSELRQGIRAVRLRNRRGELYKSVRLLIQFHFADSTA

>GmPI-PLC10

MPSKQTYSFCFCFRRRFRLPVSEAPPEIRTLFDRYSDQNGVMTATHLRSFLAEVQREDNATEEEAQTIIDGHKHLSIFHRRGLNLESFFNYLFSHHNNPPLSPSIGVHQDMSSPLSHYFIYTGHNSYLTGNQLSSDCSDIPIINALQKGVRVIELDIWPNESKDNVDVLHGRTLTSPVALIKCLRSIKQHAFVASEYPVVITLEDHLTPDLQAKVAEMITRTFGDILFAPTSKSLKEFPSPESLKRRVIISTKPPKEYLEAKEVQETEEGPQQEKPADDEEAWGKEVPSLRGGTISDYKNIEDDDVLDDEEDIDEAEKSRQDAADEYRRLIAIHAGKPKGGLTECLKVDPDKVRRLSLSELQLEKAAETHGKEIVRFTQRNILRVYPKGTRITSTNYNPLIGWMHGAQMVAFNMQGYGRSLWLMQGMFKANGGCGYVKKPDFLLKVGQNNEVFDPKAHLPVKTTLKVTIYMGEGWFHDFKHTHFDKFSPPDFYARVGIAGVPNDTVMKKTEKVEDNWSPSWNQVFEFPLAVPELALLRVEVHEYDMSEKDDFGGQTCLPVWELRSGIRAVPLYSRNGDKYANVKLLMRFEFI

>GmPI-PLC11

MASHNYKVFSCFNRKFTVTEPGPPPDVQKAFSEFADGASSLSGDHLLRFLAKHQGEVDCTAVDSERILQQSRKEDRESGLDLHDFFRFLLHDDFNSPIKSQVHHDMTAPLSHYFIYTGHNSYLTGNQLSSDCSDVPIIKALQRGVRVIELDLWPNSDEDDIEVVHGRTLTTPVSLIQCLKSIKEYAFVKSHYPLIITLEDHLTPDLQAKAAEMATQVFGELLYYPQTDSLMEFPSPESLKGRILISTKPPKEFLESKEYDDKDNEKESADELSSLPDQTSEQETDDKSAPEYKHLITIHAGKPKGDIQDELKAAGAVRRLSLSEQALEKASESYGADIVRFTQNNILRVYPKGTRLNSSNYKPHIGWTYGAQMVAFNMQGYGKSLWYMQGMFRANGRCGYVKKPEFLIEKGPHNEVFDPRRTLPVKKTLKVKVYMGTGWSLDFSQTDFDTYSPPDFYVKVCIVGVPADMAKKKTSVISNNWFPVWDEEFDFPLTVPELALLRIEVRENDKSQKDDFGGQTCLPVSELKSGFRSVPLHDEKGDKLKSVKLLMWFQFR

>GmPI-PLC12

MKTEKNSGSASKGKVRKKMETCIIKVLNFSTKKGDVYKAEPPLDLKEAFSKFARGENQMKKDQLLRFMVEHQGENISTIEDLDKIVEKFLQLGSSCSSTKTSSTRIVDVYRKQGLSLNDFIDFLLLGDFNGPLKDEVHHDMDAPLSHYFMYTGHNSYLTGNQLTSESSDEPIIEALKQGVRVIELDLWPSKSSNNDGIKVVHGRTFTTSVPVSKCLQSIKDYAFHKSDYPVILTLEDHLTPKHHDKFAKMATEIFGETLYFPETDHLTEFPSPESLKKRIIISTKPPKECRQSAIISKPVPNGREPSEEESRGLELPSSVAKLKTEDRNSSDEDQEDVNTSDNKPNQQDARQYKHLIAIHGGKSKGSMKNRLKDDIKVRRLSLSEKKLKSASESHGADLIRFTQRNILRVFPKGERVQSSNFRPYLGWLYGVQMVAFNMQGHGKSLRLMRGMFKANGGCGYVKKPEFLIRTLPHEEVFDPKKPPSVKQILKVKVYKGDGWSLDFSPTDFDRFSPPDFYTEVSIVGVPADCDKNKTRVKTDTWYPVWDEEFEFKLRVPELALLRIEVKDKDKGKKDDFAGQTCLPISELRNGFRSVPLYDRKGKKYKSVTLLMRFKLETLNKL

>GmNPC1a

MPLRRRVPLPLLLLLLLLSPAATSAVAFRKKHKIPGPIKTIVVIVMENRSFDHVLGWLKSSRPDIDGLTGTESNPLSVSSRSSPTVPVSDDALFIDSDPGHSFQAIREQIFGSNDTSAVPPPMNGFAQQAESILPGMSKTVMSGFKPQTLPVYTALANQFGLFDKWFASVPASTQPNRFYVHSATSHGAMSNVRKDLIHGFPQKTIFDSLNENNLSFGIYYQDISATLFFKSLRKLKNAVKFHDYALKFKKHAEKGKLPNYVVVEQRYFDVEVFPANDDHPSHDVAAGQMFVKEVYEVLRKSPQWEEMAVLITYDEHGGFYDHVATPVEGVPNPDGIIGPHPYYFGFDRLGVRVPTFIISPWIDKGTVIHEAEGPTPYSQYEHSSIPATVKKLFNLKSNFLTKRDAWAGTFEKYFYIRDTPRDDCPETLPDIKMLRQHGPREDSSLSEFQVELIQLASQLNGDYVLNSYPNIGKTMTVKEANRYAEDAVKRFLEAAKAALKAGANESAIVTMRPSLTSRVADGDNHKLVESY

>GmNPC1b

MSLRRGVPLSLLVLFLLLVSPAATLAFRKKHKIPGPIKTIVVIVMENRSFDHVLGWLKSSRPDIDGLTGSESNPLSVSSPSSATIPVTDDALFIDADPGHSFQAIREQIFGSNDTSAVPPPMNGFAQQAESILLGMSKTVMSGFKPHTLPVYTALANQFGLFDKWFASVPASTQPNRFYIHSATSHGAMSNVRKDLIHGFPQKTIFDSLNENGLSFGVYYQNIPATLFFKSLRKLKNAVKFHDYALKFKKHAEKGKLPNYVVVEQRYFDVEVFPANDDHPSHDVAAGQMFVKEVYEVLRKSPQWEEMAVLITYDEHGGFYDHVATPVEGVPNPDGIVGPHPYYFRFDRLGVRVPTFIISPWIDKGTVIHEAEGPTPYSQYEHSSIPATVKKLFNLKSNFLTKRDAWAGTFEKYFYIRDTPRDDCPETLPDIKMLRQHGPREDSSLSEFQVELIQLASQLNGDYVLNSYPNIGKTMTVKEA

NRYAEDAVKRFLEAAKAALKAGANESAIVTMRPSLTSRVAEGDHHKLVESY

>GmNPC2

MATQRSHHSPILFSSLILTLFVLYFPRCHHAIPNNPIKTVVVLVMENRSFDHMLGWMKRLNPAIDGVTGSESNPLSVSDPDSKRFFFRDRAHFVDPDPGHSFQAIREQIFGSNDSSLDPPPMNGFVQQAYSMDNTSHMSENVMNGFDPDLVAVYKTLVSEFAVFDRWFASVPASTQPNRLFVHSATSGGATSNVAAKLTAGYPQQTIFDSLHDAGHDFGIYYQNIPATLFYRNLRKLKYVLKFHIYDVSFKQHAKEGKLPSYTVVEQRYMDTKLLPANDDHPSHDVYEGQVFVKEVYETLRASPQWNETLFLITYDEHGGFYDHVPTPARGVPSPDGIVGPEPFNFTFNRLGVRVPTIAISPWIEKGTVVHGPNGSPSPTSEYEHSSIPATVKKLFNLPSFLTNRDAWAGTFEGIVQTRTEPRTDCPEKLPTPEKIRKGEPNEDAKLSEFQQELIQLAAVIKGDNILTSFPGTIGKDMTVKQGKYYMDDAVRSFFEAGRYARKMGVNEEHIVQMKPSLTTRSSKSPNTNP

>GmNPC4a

MASNSSTNNAGYPIKTIVVLVQENRSFDHMLGWMKSLDPKINGITGSESNPISTSNPNSNLVQFSDQSVYVDPDPGHSIQDIYEQIFGEPWSEASTAKKLPPTMQGFAQNAGRQAVPKNATATMMETVMNGFKPDLIPVYKELVKEYAVCDCWFASVPASTQPNRLYVHSATSHGLTSNDTNKLIGGLPQKTIFDSLDENGFSFGIYYQSPPATLFYRNLRKLKYVDNFRPFDLFKKHCKEGKLPNYVVIEQRFFDLLSIPGNDDHPSHDVSEGQKFVKEVYEALRGSPQWNETLFVIVYDEHGGFYDHVPTPVEGVPSPDDIVGPEPFKFQFDRLGVRIPAIIVSPWIEPGTVLHGPSGPSPTSQYEHSSIPATVKKIFNLPEFLTKRDAWAGTFEGLLTRSSPRTDCPVKLPEPVKLREAPAQEKAKLSEFQEELVQMAATLNGDHRKSIYPDKLTENMSVPDAVKYVEDAFNTFLNECEKAKQNGADESEIVDCADGCSSAPPDSKNFFYNVLSCITCNR

>GmNPC4b

MSSSSGTSATPYPIKTIVVLVQENRSFDHMLGWMKSLNREIDGVTGLESNQVSTFDPNSNRVYFGDQSGFEEPDPGHTVEDVYEQVFGEPWSESSAANKLSPRMKGFAQNSAKQKKGSTAETVMNGYKPDLLPVYKELVKEFAVCDRWFASVPGPTQPNRLYVHSATSHGLTTQDTKKLIGGLPQKTIFDSLDENGFSFGIYYQYPPSTLFFRNLRKLKYIDNFHQFDLKFKKQCKEGKLPNYVVIEQRYFDLLSLPANDDHPSHDVAEGQKFVKEVYEALRASPQWNEMLFVIIYDEHGGFYDHVPTPVDGVPSPDDIAGPEPFKFQFDRLGVRVPTIIISPWIEAGKVLHEPSGPFPTSQYEHSSIPATVKKIFNLPQFLTKRDAWAGTLEDLLSLSTPRTDCPVKLPDPVKLREAASAEQQTQLSEFQEDLIYMAATLNGDHNKSIYHKLTENLTVSEAVKYCEDAFGTFLNECEKAKQSNRIDGSEIVYCARPHTAPQSKNFWHKMLSCILCN

>GmNPC6a

MGSSKPKSSILMFVVFLCVFATAQRQQPIKTVVVLVMENRSFDHMLGWMKESINTLINGVTGDECNPVSTKSPRKDSICFTDDAEFVDPDPGHSFEDVLQQVFGSGSGSIPSMNGFVEQALSMSPNLSETVMKGFKPDSVPIYAALVKEFAVFDRWFSSIPGPTQPNRLFVYSATSHGSTSHIKRQLAKGYPQKTIFDSLHENGLDFGIYFQNIPTTLFYRNLRKLKYIWKFHQYDLKFKRDARDGKLPPLTVIEPRYFDLKGIPANDDHPSHDVAHGQMLVKEVYEALRASPQWNETLFIITYDEHGGFFDHVKTPFVNIPNPDGNTGPAPYFFKFDRLGVRVPTIMVSPWIKKGTVISGAKGPAENSEFEHSSIPATIKKMFNLSANFLTHRDAWAGTFEHVVGDLSSPRTDCPVTLPDVTPLRSTEAKENAGLSEFQSEVVQLAAVLNGDHFLSSFPDEMSKKMSVKEAHEYVRGAVSRFIRASKEAIKLGADESAIVDMRSSLTTRSSVHN

>GmNPC6b

MGSSKPRSFILLLFVFLCVFATAQEQQQPIKTVVVLVMENRSFDHMLGWMKESINTLINGVTGDECNPVSTKSPRKDSICFTDDAEFVDPDPGHSFEDVLQQVFGSSSGSGSIPSMNGFVEQALSMSSPNLSETVMKGFKPDSVPVYAALVKEFAVFDRWFSSIPGPTQPNRLFVYSATSHGSTSHIKRQLAKGYPQKTIFDSMHENGLDFGIYFQNIPTTLFYRNLRKLKYIWKFHQYDLKFKRDARDGKLPPLTVIEPRYFDLKGIPANDDHPSHDVAHGQMLVKEVYEALRASPQWNETLFVITYDEHGGFFDHVKTPFVNIPNPDGNTGPAPYFFKFDRLGVRVPTIMVSPWIKKGTVISGAKGPAENSEFEHSSIPATIKMIFNLSSNFLTHRDAWAGTFEHVVGELSSPRTDCPVTMPDVTPLRSTEAKENAGLSEFQREVVQLAAVLNGDHFLSSFPDEMSKKMSVKEAHEYVRGAVSRFIRASKEAIKLGADESAIVDMRSSLTTRSSVHN

>GhPI-PLC1

MGSYRMCVCFTRKFKVTEAAPPPDIKDAFNRYAEGGPHMTAEQLHRFLVDVQGQGLATKGDAEGIVQQLLHKRHHMAKFRRHALTLDDFHHYLFSADLNPPIGDQVHHDMTAPLSDYFIYTGHNSYLTGNQLSSDCSDVPIIKALQRGVRVVELDIWPNSTKDDVHVLHGRTLTTPVELIKCLKSIKEHAFSASPYPVVITLEDHLTPDLQAKVAQMVTQTFGKMLFCPDSECFKELPTPEKLKYRIIISTKPPKEYLEAEGNKRKMNNSHNVKESDDDVWGKEPAELTVDQEDDKTDSDASENNQDNEETDASEPEVRLSRAPAYKHLIALHAGKPKGGLKEALKVEPDKVRRLSLSEQALEKATMSHGTDVVRFTQKNILRIYPKGTRFNSSNYKPLIGWMHGAQMVAFNMQGYGRYLWLMHGMFRSNGGCGYVKKPDFLMNVNPDGSVFNPKADLPVKKTLKVKVYMGDGWHLDFKQTHFDLYSPPDFYTRVGIAGVPADEIMKKTKKKEDDWTPVWDEEFAFPLRVPELALLRVEVHEYDRSEKDDFAGQTCLPVSELKPGIRAVPLFNRKGEKFNSVRLLMRFEFI

>GhPI-PLC2

MGTYRMCGCFTRKFKIIEAAPPPDVIAAFEKYAEGGPQMTAEQLHRFLVDVQGQGGAKVSDAEEILLQVLQKRHHMAKFRKHALTLDDFHHYLFSADLNPPIDNKVHHDMTAPLSHYFIYTGHNSYLTGNQLSSDCSDVPIIKALKRGVRVVELDLWPNSTKNDVDVLHGRTLTAPVELIKCLKSIKEHAFSASEYPVVITLEDHLTPKLQAKVAQMVTQTFGKMLFRPESECLKEFPAPEDLKYKILISTKPPKEYLEAQTNKDKEKEKDSDDDVWGKEPTELTAEQEDEKTDSDASDNNQDDEDSDAFEPEVDSSRAPGYKSLIAIPGGKITGRLKEALKVEPDKVRRLSLSEQALEKATVSYGTDLVRFTQKNFLRIYPKGTRFNSSNYKPQIGWMHGAQMIAFNMQGYGRSLWLMQ

GMFRANGGCGYVKKPDFLMNVDANSERAKLPVKKRLRVKVYMGDGWHLDFKHTHFDTYSPPDFYTKVGIAGVPDDEIMKKTKIKEDNWTPVWDEEFTFPLTVPELALLRVEVHEYDMSEKDDFAGQTCLPVSELKQGIQAVPLFDRKGEKLNSTRLLMRFDFV

>GhPI-PLC3

MSKQSYRVCFCFRRRFKMTVAQAPEDVKNLFEMYSENGLMNADGLHKFLVEYQKEDETTTDDAQKIIDGSKHLPKNGLHVEAFFRHLFSDTNSPLVSLGVHHDMNAPLSHYFIFTGHNSYLTGNQLNSDCSDVPIINALKRGVRVIELDIWPNSEKDDVHVLHGGTLTAPVTLLKCLSSIKEYAFVSSDFPVVITLEDHLTPDLQAKAANMITETLGDILFTPGPEVFKEFPSLETLKKRIIISTKPPKDYTEAKEDKDKENDSKSDKADDEVAMKKAELHENKPKQKKAPEYKRLIAIHAGKPKGGLDECLEVDPEKVRRLSLSELELEKAAETHGKQIVRFTQRNMLRVYPKGIRVDSSNYNPMIAWLHGAQMVAFNMQGCDKHLWLMHGMFKGNGQCGYVKKPDFLLNTNEIFDPEVKHSEKTILKVTVYLGEGWYYDFDHTHFDSYSPPDFYVKVGIAGVPADKQMTKSKIVEDSWVPSWNQEFEFHLTMPQLALLRIKVHEYDMSKTDDFAGQTCLPVSEIRSGIRAVPLMDKKGDKYNNVKLLMRFEFTNPS

>GhPI-PLC4

MPKQSFKVCLCWRRVFKTRVVEPPLDIKNAFYSFSPSGMMTVDDLLRFLIEHQGQKNATKEDAQAIFDSLKHLNIFQRRGLHLEAFFRYLLGDLNLAHPPSKVHHDMNAPLSHYFLFTGHNSYLTGNQFSSASSVEPIKDALLRGVRVIELDLWPNSSGNDVNVCHGRTLTSSVGLQKCLEAIKENAFKASEYPVIITFEDHLNPNLQEKVAKMVTETFGDILYTSETENLEQFLSPESLKKKVLISTKPPKEYLEGNTQGEEISENEGGTVANECQSSSQWNPPEEGEHVPDEDEGKVVPEYRQLIAINARKLEGGLENWLSDDPKKVSRISLSEQKLESAAKTYGTKTVMFTQRNLLRVYPKRTRLDSSNYNPFVGWMHGAQMVAFNMQGHGKRLWIMQGMFRANGGCGYVKKPDFLLHRGPNDEVFDPNLPWDVKTIMTVKVYLGEGWHQDFHHTAFDRYSPPDFYTRIGVAGVPVDKDVKQTATIEDEWLPVWDEEFKFQLRAPELAVLRIKILDYNTTGRHYFGGQVCLPVSELRTGIRAVPLHDKKGNKYKHARLLLSIKFEAP

>GhPI-PLC5

MSKQTYRVCFCFRRRFRVAVSEAPEEIKRVFEQYSENGMMSIDALHRFLVEFQKEDKATREDAQKIVDSVKHFHRKGLNLEGFFKYLFADINPPLASLGVHHDMNAPLSHYFIHTGHNSYLTGNQLSSDCSDVPIINALKRGVRVIELDIWPNSTKDDVDVLHGRTLTTPVELIKCLRSIKEYAFVASEYPVVITLEDHLTPDLQAKVAEMVTQTFGDILFSPGSECLKEFPSPESLKGRIIISTKPPKEYLEAKEVKENENDPERVKASDEEAWGKEVPDLLKDDYKNDLGEEDEEDLDDDGDKSQHALAPEYKRLIAIHAGKPKGGLEECLRVDPDKVRRLSMSEQQLENAAITHGKEIVRFTQRNILRVYPRGTRVDSSNYNPLIGWMHGAQMVAFNMQGYGRSLWLMHGMFKANGG

CGYLKKPDFLLNPNKVFDPNVKLPVKKILKVTMYMGEGWYYDFHHTHFDAYSPPDFYARVGIAGVPFDSVMKKTKTLEDNWVPCWNEEFEFCLTVPELALLRVEVHEYDMSEKDDFGGQTCLPISELRSGIRAVPLNSRKGEKYSSVKLLMRFEFFDP

>GhPI-PLC6

MGSYRMCVCFTRKFKVTEAAPPTDVKDAFNRYAEGGPHMTAEQLHRFLVDVQGQGFATKGDAEGIVQQLLQKRHHMAKFRRHALTLDDFHHYLFSADLNPPIGDQVHHDMTAPLSDYFIYTGHNSYLTGNQLSSDCSDVPIIKALKRGVRVVELDIWPNSTKDDVHVLHGRTLTTPVELIKCLKSIKEHAFSASPYPVVITLEDHLTPDLQAKVAQMVTQTFGKMLFCPDSECLKELPTPEKLKYRIIISTKPPKEYLEAESNKRKMNNSHNVKESDDDVWGKEPAELTVDQEDEKTDSDASENNQDNEETDACEPEVRLSRAPAYKHLIAIHAGKPKGGLKEGLKVEPDKVRRLSLSEQALEKATMSHGTDVVRFTQKNILRIYPKGTRFNSSNYKPLIGWMHGAQMVAFNMQGYGRYLWLMHGMFRSNGGCGYVKKPDFLMNVNPNGSVFNPKADLPVKKTLKVKVYTGDGWHLDFKQTHFDLYSPPDFYTRVGIAGVPADEIMKKTKKKEDDWTPVWDEEFVFPLRVPELALLRVEVHEYDMSERDDFAGQTCLPVSELKPGIRAVPLFNRKGEKFNSVRLLMRFEFI

>GhPI-PLC7

MGYMGDYSMCICFPKKFGVTEAGPPVDVKEVFMKYATGGGSGMTVEQLRRFLVEVQGDVEASMEDAERIVEEVFKRRHNNVKLPELALSLEDFQFYLFCVDLNPPLLNKVHQDMTAPLSHYFIYTGHNSYLTGNQISSDCSDVPIIKALKRGLRVVELDLWPNSSKDDVLVLHGWTLTTPVELIKCLRSIKEHAFSASPYPVIITFEDHLTPDLQAKVAQMVIQTFGNMLFCPPESDCVKEFPSPEELKYRIVISTKPPKEYLEDKNLSSRGSNSLKDKDSDEDTWGRMSADLTNDDDKSDCDASEHSQCDGDNEACDQLLRPLGAPAYKNLISIPAGKPKGKLREKLKVEKDKVRRLSLSEQKFEKATVCHGTDVVRFTQRNILRIYPKGTRVNSSNYNPLIGWMHGAQMVALNMQGYGKPLWLMHGMFGSNGGCGYVKKPDFLMNVGPNDQVFDAKAKLPVKKILKVKVHMGDGWHLDFKQRYLNLWSSPEFYTRVGIAGVPADKTMKKTKKRKGNWTRVWDEEFTFQLTVPEIALLRIEVHEYNMSEKDDFAGQTCLPMSELRPGFRAVPLFNRKGEKYTSLRLLLRFEFVQVDI

>GhPI-PLC8

MSKQSYRVCLCFRRRFKMAVAQAPEDVKNLFEMYSENGLMNVDGLHKFLVEYQKEDETTTDDAQKIIDGSKHLPKNGLHVEAFFRHLFSDTNSPQVSLGVHHDMNAPLSHYFIFTGHNSYLTGNQLNSDCSDVPIINALKRGVRVIELDIWRNSEKDDVHVLHGGTLTAPVTLLKCLSSIKEYAFVSSDFPVVITLEDHLTPDLQAKAANMITQTLGDILFTPGSEVFKEFPSPETLKKRIIISTKPPKDYTEAKEDKDKENDLKSDKADDEVATKKAEPHENKPKQKKAPEYKRLIAIHAGKPKGGLDECLEVDPEKVRRLSLSELELEKAAETHGKQIVRFTQRNMLRVYPKGIRVDSSNYNPMIAWLHGAQMVAFNMQGCDKHLWLMHGMFKANGQCGYVKKPDFLLNTNEIFDPEV

KHSEKTILKVTVYLGEGWYYDFDHTHFDPYSPPDFYVKVGIAGVPADKQMTKSKIVEDSWVPSWNQEFEFHLTMPQLALLRIEVHEYDMSEKDDFAGQTCLPVSEIRSGIRAVPLMDKKGDKYNNVKLLMRFEFTNPS

>GhPI-PLC9

MPKQNFKVCLCWRRIFKTRVVEPPPDVKNAFNHFSQSGTMTVDDLLTFLIEHQGENNATKEDAQAIFDSLKHLNIFHRRGLHLEAFFRYLLGDHNLAHPPSSKVHHDMTAPLAHYFLFTGHNSYLTGNQVISASSVEPIKDALLRGVRVIELDLWPNSKGDDVEIRHGGTLTSPVDLQKCLQAIKENAFHASEYPVVITFEDHLNPNLQKKVAKMVTETFGDMLYWSETENMQQFPSPESLKKRILISTKPPKEYLGENRGDVSETESGRVRNELSRNPSDVGEHFPDEDEENTVVQYRQLIAIHAGKLKGGLENWLSDDPMKVRRLSLSEQELENAIRTYATKIVRFTQRNLLRVYPKGTRLDSSNYNPFVGWMHGAQMVAFNMQGYGKYLWIMQGMFKANGGCGYVKKPDFLLRRGENDEVFNPSAPLEVKTVMRVKVILGEGWHQDFHHTAFDRYSPPDFYTKIGIAGVPEDKDVKQTAIIEDEWLPVWDQDFEFLIRVPELAVLRIQVLEYDTTGRPDFGGQTSLPVSELRTGIRTVPLCDKKGNKYKHVRLLLSINFGRPYDL

>GhPI-PLC10

MGKEKEREKKSSSLESGSYNYKMFSFFNRKFKINEVEPPSDVNKAFSLFTDDGSTHMTAEQLRRFMSVHQCEVSTRLEDAQNIIEQVVNRRHHITKFARHTLNIEDFFYFLLSDDLNGPIRTQVHHDMSAPLSHYFIYTGHNSYLTGNQLSSDCSEVPIIKALQNGVRVIELDLWPSKDEILVLHGRTLTTPVSFIQCLTSIKEYAFVSSPYPVIITLEDHLTPELQAKAADMITQTFETMLYYPESDLTEFPSPESLKYRIMISTKPPKEYLEVRSKDASEDESSPKDDSDVSESDQEDEDFKSLQAGVSGYKRLITIHAGKPKGSLKIALKEVTDQVRRLSLSEHQLEKLAGSHGLDIVRFTQRNILRVYPKGTRFTSSNYKPTIGWMHGAQMVAFNMQGYGKSLWLMHGMFRANGGCGYVIKPDILTRSADELFDPKATLLPVQKTLKVKIYMGDGWRLDFKHTHFDAYSPPDFYTK

IYIVGVPADEAKKKTKIIEDDWCPVWDEEFSFPLTVPELALLRIEVREYDISEKDDFGGQTCLPVPELRTGFRSVPLHDKKGVKHKNVRLLMRFEFV

>GhPI-PLC11

MSKQTYRVCFCFRRRFRVAVSEAPEEIKQVFEQYSEHGMMSIDGLHRFLVEFQKENKATREDAQKIVDSVKHFHRKGLNLEGFFKYLFGDINPPLASLGVHHDMNAPLSHYFIHTGHNSYLTGNQLSSDCSDVPIIHALKRGVRVIELDIWPNSTKDNVDVLHGRTLTTPVELIKCLKSIKDYAFVASEYPVVITLEDHLTPDLQAKVAEMVTQTFGDILFSPGSECLKEFPSPESLKGRIIISTKPPKEYLEAKEVKENENNSERAKASDEEAWGKEVPDLLKDDDKNDLGEEDEEDPDEDGDKPQHALAAEYKRLIAIHAGKPKGGLEECLKVDPDKVRRLSMSEQQLEKAAITHGKEIVRFTQRNILRVYPRGTRVDSSNYNPLIGWMHGAQMVAFNMQGYGRSLWLMHGMFKANGGCGYLKKPDFLLNTKAVFDPTVKLPVKKILKVTMYMGEGWYYDFHHTHFDAYSPPDFYARVGIAGVPFDSVMKKTKTLEDNWVPCWNEEFEFHLTVPELALLRVEVHEYDMSEKDDFGGQTCLPISELRSGIRAVPLNSRKGEKYSSVKLLMRFEFIDP

>GhPI-PLC12

MSKQTYRVCFCFQRRFRLAVSEAPEDIKKLFEQYSENGIMTIDGLQRFLVEVQKEDKATREDAQKIIDSVKHFHRKGVNLEAFFKYLFGDINPPLASLGVHHDMSAPLSHYFIYTGHNSYLTGNQLSSECSDVPIINALKRGVRVIELDIWPNSTKDDVDVLHGGTLTTPVELIKCLRSIKEYAFVASEYPVVITLEDHLTPKLQAKVAEMVTQTFGEILFSPGPECFKEFPCPESLKRRIIISTKPPKEYLEAKEVKDKEDDSQRGKASDEEAWGNEVPDLKGSHVANYKNDFDEEDEEDTDDGEKSQHSLAPEYKHLIGILAGKPKGGFDSWLRVDPDKVTRLSMSEQKFEKAVVTHGKQIVRFTRQNVLRVYPKSTRFDSSNYNPLIGWMHGVQMVAFNMQGHGRSLWLMHGMFKANGGCGYVKKPDFLLNSMEVFDPKIKLPVKTTLKVTVYMGEGWYYDFHHTHFDAYSPPDFYTRVGIAGVPADSVMKKTKILEDNWLPSWNEVFEFPLTVPELALLRIEVHEYDMSEKDDFGGQTCLPISELRSGIRAVPLYSRKGEKYNSVKLLMHFEFI

>GhNPC1a

MENRSFDHLLGWLKSTRPDIDGLSGTESNPVNVADPNSPFISVSDDALFVDSDPGHSFQAIREQIFGSNDSSADSAPMNGFAQQAESMGEGMGRTVMSGFKPSRLPVYTKLANEFGVFDRWFASVPASTQPNRFYVHSATSFGATSNVKKDLIHGFPQKTIFDSLDENGLSFGIYYQNIPATLFFKSLRKLKFLTKFHNYALKFRLHARLGKLPNYVVVEQRYFDVKEFPANDDHPSHDVARGQRFVKEVYEILRSSPQWKEMALLITYDEHGGFYDHVPTPVSGVPNPDGIVGPDPFYFKFNRLGVRVPTLLVSPWIDKATVIHEPTGPTPSSQFEHSSIPATVKKLFNLNSNFLTKRDAWAATFENYFKLRTTPRTDCPETLPEVTTSLRPWGPKEDASLSEFQVELVQLASQLNGDYVLNTYPYIGKSMRVGEANRYVEDAVKRFLEAGKAAIRAGANESAIVTMRPSLTSRIEDRGQHVEAY

>GhNPC1b

MENRSFDHLLGWLKSTRPDIDGLSGTESNPVNVADPNSPFISVSDDALFVDSDPGHSFQAIREQIFGSNDSSADSAPMNGFAQQAESMGEGMGRTVMSGFKPSRLPVYTKLANEFGVLDRWFASVPASTQPNRFYVHSATSFGATSNVKKDLIHGFPQKTIFDSLDENGLSFGIYYQNIPATLFFKSLRKLKFLTKFHNYALKFRLHARLGKLPNYVVVEQRYFDVKEFPANDDHPSHDVARGQRFVKEVYEILRSSPQWKEMALLITYDEHGGFYDHVPTPVSGVPNPDGIIGPDPFYFKFNRLGVRVPTLLVSRWIDKATVIHEPTGPTPSSQFEHSSIPATVKKLFNLNSNFLTKRDAWAATFENYFKLRTTPRTDCPETLPEVTTSLRPWGPQEDASLSEFQVELVQLASQLNGDYVLNTYPSIGKSMRVGEANRYVEDAVKRFLEAGKAAIRAGANESAIVTMRPSLTSRIEDRSQHVEAY

>GhNPC2a

MFKPANTAIFFFFVLFNSFSCHGGPVKTIVVLVMENRSFDHMLGWMKKINPQINGVDGTEWNPLSTTDPNSKKLFFQNQAQFVDPDPGHSFQAIREQIFGSNDTSANPPPMNGFAQQAYSMDLSTTMSQNVMNGFDPEMVAVYKSLVSEFAVFDRWFASVPSSTQPNRLYVHSATSAGATSNIPALLVKGYPQRTIFENLDDAGISWGIYYQNIPATLFYKNLRKLKYLFRFRPYGVTFKKHAQEGKLPGYVVVEQRYMDTKLEPANDDHPSHDVYQGQMFVKEVYETLRASPQWNQTLLIITYDEHGGFYDHVATPVTGVPSPDGIVGPEPFFFHFDRLGVRVPTIMVSPWIDKGTVVHGANGRPFPTSEFEHSSIPATVKLLFNLTSPFLTKRDEWAATFESILRTRSDPRTDCPETLPTPARIRRGEAIEEAKLSEFQQELVQLAAVLKGDHILTSYPERIGKDMSVKEGKEYMEDAVKRFFEAGHYAKKMGVDGEQIVQMKPSLTTRSSKPSSQHP

>GhNPC2b

MFKPASTAIFFFFFSCHGGPIKTIVVLVMENRSFDHMLGWMKKINPEINGVDGTEWNPLSTTDPNSKKLFFQNQAQFVDPDPGHSFQAIREQIFGSNDTSTNPPPMNGFAQQAYSMDPSTTMSQNVMNGFDPEMVPVYKSLVSEFAVFDRWFASVPSSTQPNRLYVHSATSAGATSNIPALLVKGYPQRTIFENLDAAGISWGIYYQNIPATLFYKNLRKLKYLFRFRPYGVTFKKHAQEGKLPGYVVVEQRYMDTKLEPANDDHPSHDVYQGQMFVKEVYETLRASPQWNQTLLIITYDEHGGFYDHVATPVTGVPSPDGIVGPEPFFFHFDRLGVRVPTIMVSSWIDKGTVVHGANGRPFPTSEFEHSSIPATVKLLFNLTSPFLTKRDEWAATFESILRTRSDPRTDCPETLPTPARIRRGEANEEAKPSEFQQELVQLAAVLKGDYILTSYPERIGKEMSVKEGKEYMEDAVKRFFEAGHFAKKMGVDGEHIVQMKPSLTTRSSKPSSQHP

>GhNPC3

MAVETSSATPSPVKTVVVLVQENRSFDHMLGWFKTINPEIDGVTGSESNPISTSDPNSTQITFKDTAGYVDPDPDHSFQAIYEQVSGKTWDTSNPDPNPGIKMNGFVQNAERTTPGLSETVMNGFKPEAVPVFKQLVTEFAVCDRWFASLPASTQPNRLYVHSATSHGAMSNNTQQLIEGFPQKTIFESLEENGYSFGIYYQSFPSTLFYRKLRHLKYVDNFHQYDLSFKRHCKDGKLPNYVVIEPRYFDILTAAANDDHPSHDVSEGQKLVKEIYEALRSSPQWNEILFLVIYDEHGGFYDHVPTPTGVPSPDDIVGPEPYNFKFDRLGCRVPAIMVSPWIEPGTVLHRPSGPDPTSEFEHSSIAATLKKIFNLKEFLTKRDAWAGSFDIVVNRSTPRTDCPEKLAEPVKMRDSDAKETAKLSDFQEELVQAAAALKGDPFNLVENMTVSSGLKYVEDAFKKFYDDGQKAKEINEVEDTVSADASTRRTTASKTFMQKVFSCLVCDR

>GhNPC4

MYVYISQPIPNQQRTYKNRTMVSQGSNSASSYPIKTIVILVQENRSFDHMLGWFKSLNPEIDGVTGSESNPISTSDPNSPMVFFKDNSEYVDPDPAHSIQAIYEQVFGHPWSSDLPNPPHEPTMNGFAQNAERTEKGMAEAVMKGFKPDAVPVYKELASKFGICDRWFASVPASTQPNRMFVHSATSYGQESNDAIKLIKGFPQKTIFESLDESGFSFGIYYQYPPSTLFFRNLRQMKYLKNFHQFDLHFKKHCEEGKLPNYVVVEQRYFDLLSVPANDDHPSHDVSEGQKFVKQVYEALRSSPQWKEMLLVITYDEHGGFYDHVPTPTNGVPSPDDIVGPEPYHFKFDRLGVRVPTFFVSPWIEPGTGKSRESPLLLSFNLGYLDHVCMVMEFAVIHRPLGPYPTSQFEHSSIPATVKKIFNLKEFLTKRDAWAATFEGVINRKNPRVDCPVTLPEPVKMRPTEAKETAKLSDFQKELVQMAAVLNGDHKSDMYPHKLVEKMTVAEAAKYVNGAFNKFCDECQRGGIHESEIVELGKQVERPKGRSFIYKFFKCLVCHD

>GhNPC6a

MGESKASPPPSFSFIFSLFLTVACLFTPLGAQQQSPIKTIVVLVMENRSFDHMLGWMKQHVNPSINGVTGDECNPVSTKNPNPESICFTDDAEFVDPDPGHSFEAVEQQVFGSSTIPSMSGFVEQALSMSKNLSETVMKGFRPESVPVYAALVKEFAVFDRWFSSIPGPTQPNRLFVYSATSHGSTSHVKKQLAHGYPQKTIFDSLHENDKDFGVYFQNIPTTLFYRNLRKLKYVFKFHQFDLKFKKDARKGKLPSLTVIEPRYFDLKGLPANDDHPSHDVANGQKLVKEVYEILRASPQWNQTLLVITYDEHGGFYDHVHTPYINVPSPDGNTGPAPSFFKFDRLGVRVPTIMVSPWIKKGTVISGPKGPFPNSEFEHSSIPATIKKMFNLSSNFLTHRDAWAGTFERVVGELSSPRTDCPEKLPEAAPLRTTAANEDAGLSEFQSEVVQLASVLNGDHFLSGFAEEMHTKMSVKGAHE

YVKGAVSRFIRASKEAIKLGADESAIVDMRSSLTTRSSSIHN

>GhNPC6b

MGESKTSPPPSFSFIFSLFLTVACLFTPLGAQQQSPIKTIVVLVMENRSFDHMLGWMKQHVNPSINGVTGDECNPVSTKNPNPESICFTDDAEFVDPDPGHSFEAVEQQVFGSSTIPSMSGFVEQALSMSKNLSETVMKGFRPESVPVYAALMKEFAVFDRWFSSIPGPTQPNRLFVYSATSHGSTSHVKKQLAHGYPQKTIFDSLHENGKDFGVYFQNIPTTLFYRSLRKLKYVFKFHQFDLKFKKDARKGKLPSLTVIEPRYFDLKGLPANDDHPSHDVANGQKLVKEVYEILRASPQWNQTLLVITYDEHGGFYDHVHTPYINVPSPDGNTGPAPSFFKFDRLGVRVPTIMVSPWIKKGTVISGPKGPFPNSEFEHLSIPATIKKMFNLSSNFLTHRDAWAGTFEHVVGELSSPRTDCPEKLPEAALLRTTEANEDAGLSEFQSEVVQLASVLNGDHFLSSFAEEMRTKMSVKGAHEYVKGAVSRFIRASKEAIKLGANESTIVDMRSSLTTRSSSIHN

>GhNPC6c

MEGSFSFIFLLFLLPFVVSQGSPIKTIVVLVMENRSFDHMVGWMKQSINPTINGVTGNECNPISTKTPNPKSICFTNDAQFVDPDPGHSFEAVEQQVFGSTLSSFPSMSGFVEQAFSISPNMSETVMKGFKPEAVPIYATLVKEFAVFDRWFSSIPGPTQPNRLFVYSATSHGSTSHVKKQLAQGYPQKTIFDSLHENGKDFGVYFQNIPTTLFYRNLRKLKYVFKFHQFDLKFKKDALNGKLPSLSVIEPRYFDLKGLPANDDHPSHDVANGQKLVKEVYETLRASPQWNETLLVITYDEHGGFYDHVKTPFVNVPNPDGNTGPAPSFFKFDRLGVRVPTIMVSPWIKKGTVISGPKGPTPNSEFEHSSIPATIKKIFNLSSNFLTHRDAWAGTFEDVVSHLTSPRTDCPETLPDVVPLRATEAKEDAALSEFQSEVVQLAAVLNGDHFLSSFPDEMSKKMTVKEAHEYTKGAISRFIRASKEALKLGAAESAIVDMRSSLTTRSSNP

>GhNPC6d

MERSFSFIFLLFILPFVVSQESPIKTIVVLVMENRSFDHMVGWMKQGINPTINGVTGNECNPISTKTPNPKSICFTNDAQFVDPDPGHSFEAVEQQVFGSTPSSFPSMSGFVEQAFSISPNMSETVMKGFRPEAVPIYASLVKEFAVFDRWFSSIPGPTQPNRLFVYSATSHGSTSHVKKQLAQGYPQKTIFDSLHENGKDFGVYFQNIPTTLFYRNLRKLKYVFKFHQFDLKFKKDALNGKLPSLSVIEPRYFDLKGLPANDDHPSHDVANGQKLVKEVYETLRASPQWNETLLVITYDEHGGFYDHVKTPFVNVPNPDGNTGPAPSFFKFDRLGVRVPTIMVSPWIKKGTVISGPKGPTPNSEFEHSSIPATIKKIFNLSSNFLTHRDAWAGTFEDVVSHLTSPRTDCPETLPDVVPLRTTEAKEDAALSEFQSEVVQLAAVLNGDHFLSSFPDEMSKKMTVKEAHEYTKGAVSRFIRASKEALKLGAAESAIVDMRSSLTTRSSNP

>SbNPC1

MVAAARRDPGTRLLVALLLLALVVSGHCLDAHHRGLKRRRRKHEIHSPVKTVVVVVMENRSFDHILGWLSRTRPDIDGLTGRESNRLNASDPSSPEIFVTDEAGYVDSDPGHGFEDIREQIFGSADTSAVPPPMSGFAQNARGMGLGMPQNVMSGFKPDAVPVYASLADEFAVFDRWFASVPTSTQPNRLFVHSATSHGLTFNARKDLIHGFPQKTIFDSLEENGLSFGIYYQNIPATLFYQSLRRLKHLVKFHQYSLKFKLHAKLGKLPNYVVIEQRYFDCEMFPANDDHPSHDVARGQRFVKEVYETLRASPQWNETALIITYDEHGGFYDHVPTPVVGVPQPDGIVGPDPYYFKFERLGVRVPTFLISPWIEKGTVIHAPNGPQETSQYEHSSIPATVKKLFNLHSNFLTKRDAWAGTFENYLKIRKTPRTDCPEKLPEVTKSLRPFGPKEDSSLSEFQVELIQLASQLNGDHVLNTYPDIGRTMTVGKANRYAEDAVARFLEAGRIALRAGANESALVTMRPALTSRASLSSGLSS

EL

>SbNPC2

MAAARARPPPVAALLLLVLLLTGAAGSSTTTSTTSPIKTVVVLVMENRSFDHMLGWMKRLNPEIDGVTGREWNPANTSDPSSGRVYFGDGAAYVDPDPGHSFQEIRQQIFGSDDASGPARMDGFVQQAASIGGGNMTDAVMHGFAPDSVAVYRELVSQFAVCDRWFASVPSSTQPNRLFVHSGTSGGATSNNPTLLAEGYPQRTIFDNLHDAGLSFGVYFQDVPAVLFYRNLRKLKYLLDFHPLRPSFADHARRGVLPNYAVIEQHYLDSKLDPANDDHPSHDVYQGQMLVKYVYETLRASPQWNQTLLVITYDEHGGFFDHVPTPVAGVPSPDGIVGPPPYNFTFDRLGVRVPAILVSPWIDKGTVVHAPTGPTPTSQYEHSSIPATVKKIFNLPQGFLTKRDAWAGTFEGVVQKRTEPRTDCPEQLPTPTRIRQTEADEEAKLSEFQQEIIQLASVLNGDHQLASLQDRIRDEMNVREGIDYMKAAVKRYFEAGASARRMGVDGEQIVKMRPSLTTRIQRP

>SbNPC3a

MAEAPAAATGNSSNNHKIKTVVVVVQENRSFDHMLGWMKTLNPDIDGVTGVETNHVDASDPTSRAVRFSDGAEYVDPDPGHSMQAIYEQVYGTPFVDAATTPITPPGVPAAPPMSGFAQQAEKEKPGMSGTVMSGFRPDAVPVYRELVKEFAVCDRWFASNPASTQPNRLFVHSATSHGLVSNDTKALVAGLPQRTIFDALHDEGFSFGIYYQYPPSTLFYRNLRQLKYVGSFHAFDLDFRRHCREGKLPNYVVVEQRYFDLEILPGNDDHPSHDVAEGQRFIKEVYEALRSGPQWEETLLVVTYDEHGGFYDHVPTPAGAGVVPSPDGIVSASPFFFGFDRLGVRVPALLVSPWIEPGTVLHGPSGPYPTSEFEHSSIPATVKKLFNLRSFLTKRDAWAGTFDCVLTRDTPRTDCPRTLPEPVKLRRTVAAEHAPLSEFQEELVQLAAVLNGDHTKDSYPHKLVEGMTVAEAARYCVDAFKAFRDECEKCKKRGEDGSHIPTVKPSASGKDKDKSKSKSKSSFVSKALLACLPCARPSS

>SbNPC3b

MADKIKTVVVLVQENRSFDHMLGWMKSLNSEIDGVTGAEVNYTVAGDATSTAVHFGNASQYVDPDPGHSFMAIYEQIYGDAFTWGVTAPATKPGVTVPPMSGFAQQAEAEKPGTPHAVMNGFRPDAVPVYRELVGEFAVCDRWFASVPTSTQPNRMFVHSATSHGLVGNDKKLLREGMPQRTIFDALHDAGHSFGIYYQFPPAVLLYRNMRQLKYIGKFHEYELHFKRHCREGKLPNYVVIEQRYLDWKLLPGNDDHPSHDVAHGQRLVKEVYEALRSSPQWNEILFVITYDEHGGFFDHVPTPVDGVPSPDGIVSAAPINFAFDRLGVRVPAMFISPWIEPGTVIHRPPSGPEPTSQYEHSSIPATVKKIFGLKEFLTKRDAWAGTFEHVLTRATPRTDCPETLPEPVRLREAKAEEDQRREVSEFQAELVQLGAALNGDHATEAYESDKLVKGMTVAEASDYCRAAFARFREECQRCHEGGMDECHVPALPPPTASKLCGCLPCFSAS

>SbNPC6

MHPWPTIQRTVTRRMPPKHASPCPPSVPAPSSSTCTSTSTAGISSNTPVPDMATRSTHRRPLLLILLFLLLFAAVNGSARPSTSPIKNVVVLALENRSFDHMLGWMRRLLGLPVDGLTGAECNPNSTNSTTSSICVSADADLVVPDDPGHSFEDVLEQVFGNGNISAAQPSMSGFVRSALSVNALLSSAVMRAFRPSLLPTFSALAPAFAVFDRWFSSIPGPTQPNRLFLYSATSRGAVAHDKLDLLLGYPQRTIFESLAADGHDFAVYFKTIPTVLFYRRLRALRYAARSFHRYDAAFKDHARRGVLPALSVIEPRYFDLTGTPADDDHPAHDVANGQRLVKDVYEALRASPQWNQTLLIVTYDEHGGFYDHVSTPTAGVPSPDGIRGPPPFFFKFDRLGVRVPTIMVSPWIKKGTVVGRAVGPTDTSEFEHSSIPATIKKIFNLSSDFLTKRDAWAGTFEHIFTELDQPRTDCPETLPEVPFVRPTPPKEHGWLSDFQRELVELASFLNGDYMLTSLAQESRKKKMTVKQADAYVRRAITSFLQASKQAVRLGANESAIVTMRSSLTSKSSSSSSP

>SbPI-PLC1

MGTTYKCCLIFKRRFHARDAPPPEDVRALFSLHTGGGTHMGADGLRRYLDATQEEASRLDDAEVERLLEQIRLQQHQGGHRARLPRLARPLLALDDFHRYLFSHDLNPPLRHHQVHHDMTRPLSHYFIYTGHNSYLTGNQLSSDCSDVPIIKALQRGVRVIELDMWPNSTKDDINILHGRTLTTPVSLIKCLISIKEYAFVASPYPVIITLEDHLPSELQEKVAKMVLDVFGDILYYPPDSDHLKEFPSPEQLKGRVLLSTKPPKEYLEAKADDTMKEGDADLHLAKGANDDAAWGKEVPDFQTEIQSAKKHDDDAPGHQREDDDDDDDEEEEQKMQPHLAPQYKHLITIRAGKPKGSLADALKSDPEKVRRLSLSEQQLAKVAEDHATEIVRFTQRNILRIYPKGTRVTSSNYNPFIGWVHGAQMVAFNMQGYGRALWLMHGFYKANGGCGYVKKPDFLMQTEPQVFDPREPQPVKKTLKVKVYMGDGWRMDFKQTHFDQYSPPDFYTRVGIAGVPADSVMKKTKAIEDNWMPVWEEEFSFPLTVPEIALLRVEVHEYDMSEKDDFGGQTVLPVSELRPGIRAVALFDRKGNKYNNVKLLMRFEFA

>SbPI-PLC2

MPKRDKAASPRASPPHTPPPGTDSEAEEADQAGASAAEMGTYKCCIFFTRRFALADTSTPEDVRALFSRYAGGSPYMLADDLRRYLAAWGGADGEVPEQIIDRILQDRSRTPRFGRPALTVDDFLHFLFSEDLNPPLRHSKVNQDMNAPLSHYFIYTGHNSYLTGNQLSSDCSDVPIIKALQIGVRVIELDIWPNSSKDDIDVLHGRTLTAPVSLIKCLRSIKEYAFVASPYPVIITLEDHLTPDLQAKVAKMVLEVFGDILYYPESKHLQEFPSPEALKGRVMLSTKPPKEYLEAKGGTMKEREIEAQFKKGEREEAAWGVEVPDIHEEMQVANRNDDDLLYRERGADGDDEKKTKHVAAEYKHLITIKAGKPKGPLVDALKNDPDKVRRLSLSEQELAKVAARHGPNIVSFTHRNMLRIYPKGTRFNSSNYNPFLGWVHGAQMVAFNMQGHGRALWLMHGFYKANGCCGYVKKPDFLMQTCPDGKVFDPEADLPVKATLKVKVYMGEGWQKDFKQTHFDTYSPPDFYVKVGIAGVPLDSVMRKTKAVEDSWVPVWEEEFTFPLTVPEIAVLRVEVHEQDVSEDDFGGQTALPVAELRPGIRAVPLFDHKGLKFRSVKLLMCFEFA

>SbPI-PLC3a

MGSYKYKYCMCFTRKFRSPDAQPPPDVRAAYLSFNSDVHALRRFLSQAQAEHPADVDRILALLTAASGGHGIARLVTRSPAPAMPTLEDFFAFLFSPELNPPLPHQVHQDMSAPLSHYFIFTGHNSYLTGNQLNSDSSDIPIIKALQRGVRVIELDMWPNSSKTNVDILHGGTLTAPVEMIRCLKSIKEYAFCASPYPLVITLEDHLTPDLQAKVAKMLTETFGDLLFVPSSDPMKEFPSPAALMKRIIISTKPPQEYKEFLKVKDNQNGSGNIADLPDTGSLRRVDSNADNQNGSGNLAADMGSLRRIDSNADESDGKDELDELDEEDSDEDDPKFQQDTACEYRKLITIQAGKPKGHLRDALKVDPEKVRRLSLSETQLAKATTSHGAEVIRFTQNNILRVYPKGTRVNSSNYDPMNAWTHGAQMVAFNMQGHDKALRLMQGFFRANGGCGYVKKPDFLLRTGPNGEVFDPKGSLPVKKTLKVKVYMGDGWRMDFSKTHFDAFSPPDFYTRVGIAGVKADTVMKKTRVIEDQWVPVWDEEFTFPLKVPELALLRIEVQEYDMSEKHDFGGQTCLPVWELKQGIRAVPLHDRKGNRYKSVRLLMRFDFF

>SbPI-PLC3b

MGSYKYKYCMCFTRKFRSPDAQPPPDVRAAYLSFASDVHALRRFLSQAQAEHPADVDRILALLTAASVGHGIARLVTRSPAPAMPTLDDFFAFLFSPELNPPMGPPHQVHQDMSAPFSHYFVFTGHNSYLTGNQLNSDSSDVPIIKALQRGVRVIELDMWPNSSKTNVDILHGGTLTAPVEMVRCLKSIKEYAFCASPYPLVITLEDHLTPDLQAKVAKMLTETFGDLLFIPSSDPMKEFPSPAALMKRIIISTKPPQEYKEFLKVKDNQNGSGNIADLPDTGSLRRIDSNADNQNGSGNLAADTGSLRRIDSNADESDGKDELDEQDEEDSDEDDPKFQQDTACEYRKLITIQAGKPKGHLRDALKVDPEKVRRLSLSETQLAKATTSHGAEVIRFTQNNILRVYPKGTRVNSSNYDPMNAWTHGAQMVAFNMQGHDKALRLMQGFFRANGGCGYVKKPDFLLTTGPKGEVFDPKGSLPVKKTLKVKVYMGDGWRMDFSKTHFDAFSPPDFYTRVGIAGAKEDTVMKKTKVIEDQWVPVWDEEFTFPLRVPELALLRIEVQEYDMSEKHDFGGQTCLPVWELKKGIRAVPLHDRKGNRYKSVRLLMRFDFV

>SbPI-PLC4

MTTTYRVCCFLRRFRAASNEPSEAVRDVFQAYADGGGVVGEEALRRLLREVQGETEAGADAAAKEVMAFAAEQRLLKKGGLTAEGFHRWLFSDANAALDPRRGVYQDMGLPLSHYFIYTGHNSYLTGNQLSSGCSERPIVKALHDGVRVIELDLWPNAGKDDVEVLHGRTWTSPVELIKCLEAIKEHAFVTSPFPVILTLEDHLTPDLQANVAKMLKETFGEMLYVSESEKMAEFPSPDELKGKIIISTKAPKEYLQTKSGKEEAEEGVWGEEISDDKTTAHQMSEQFSGKYSAAAEEEAAAGAEEEAAEAEAEKKARQGTDNEYRRLIAIQLTRRKHDMEQDLRVDPDKVTRLSLGEKAFEKAIVSHGDHIVRFTQRNLLRIFPRSTRITSSNYNPLMGWRYGVQMVAANMQGHGRKLWLTQGMFRANGGCGYVKKPDILMNSAAADDPAGGIGKLFDPTRADLPVKTRLKVTVYMGDGWRFDFRKTHFDRCSPPDFYVRVGIAGVAADMRMEQTRVVMDSWIPAWDHEFGEFPLAAPELALLRVEVHESDNHQKDDFGGQTCLPVWELRPGIRSVRLADHKGQPLRSVKLLMRFEFFSSP
