# Supplementary figures and images for: Genome-Wide Investigation of the Phospholipase C Gene Family in Zea mays
Source: Front Genet. 2021 Jan 12;11:611414. doi: 10.3389/fgene.2020.611414 (PMC7835795; doi:10.3389/fgene.2020.611414)

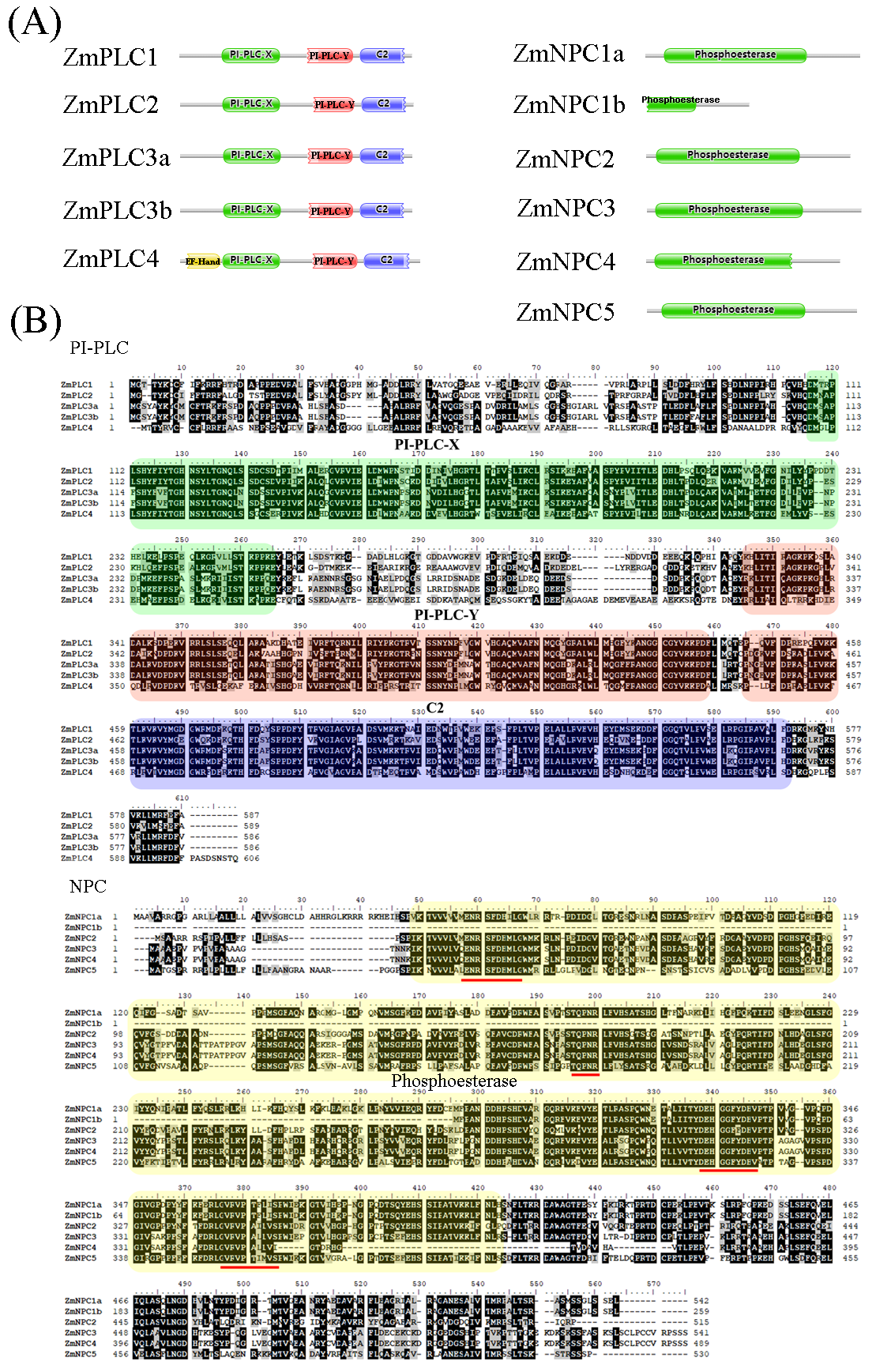

Supplement: Supplementary Figure 1 — Schematic domain structures of ZmPLC (A) and Multiple sequence alignments of ZmPLCs (B). Protein sequences of ZmPLC were aligned by Bioedit. The conserved and characteristic domains and sequences of PI-PLCs and NPCs have been marked with colored box and red underline, respectively. [file Image_1.TIF]
