# Supplementary material for: Genome-Wide Investigation of the Phospholipase C Gene Family in Zea mays
Source: Front Genet. 2021 Jan 12;11:611414. doi: 10.3389/fgene.2020.611414 (PMC7835795; doi:10.3389/fgene.2020.611414)
Supplement: Supplementary Table 1 — Primers and their sequences used in this study. [file Table_1.DOCX]

Table S1 The primers used for real-time quantitative RT-PCR (qRT-PCR)

| gene | Primer name | Primer sequence |
| --- | --- | --- |
| *ZmPLC1* | F  R | AAAGGACGACGAGAACGAC  TCAAACTGAGACGCCGAAC |
| *ZmPLC2* | F  R | AGCAGATCATCGACCGTAT  ATGGACCTTGGAGTAGCG |
| *ZmPLC3a* | F  R | TGGAGGGACATTGACTGC  ATCGGGTCTGGATTAGGG |
| *ZmPLC3b* | F  R | AGCTGAGTTGCCAGACCA  AGGCTGTATCCTGCTGAAAT |
| *ZmPLC4* | F  R | CCAGACGGAAGCACGACATAGA  GCGGGTTGTAGTTGGACGAAG |
| *ZmNPC1a* | F  R | TCCGCAGTGGAACGAGAC  GGTGAGATGAGAAAGGTGGG |
| *ZmNPC1b* | F  R | CAGCCTGATGGAATAGTTGG  ATCCTGTGGACCGTTTGG |
| *ZmNPC2* | F  R | CCCGTTCTCCTCTTCTTCCTCC  GGTTCAGCCGCTTCATCCAC |
| *ZmNPC3* | F  R | TCTTGGACGAGTGCGAGAAATG  AGCAGGGAAGGCAGGACAGC |
| *ZmNPC4* | F  R | TCTTGGACGAGTGCGAGAAATG  AGCAGGGAAGGCAGGACAGC |
| *ZmNPC5* | F  R | ATGGCAACCGGATCCCCTCGCCGCC  GGCGGCGAGGGGATCCGGTTGCCAT |
| *ZmTUB* | F  R | CTACCTCACGGCATCTGCTATGT  GTCACACACACTCGACTTCACG |
